# Cumulative Effects of Non-Aflatoxigenic *Aspergillus flavus* Volatile Organic Compounds to Abate Toxin Production by Mycotoxigenic Aspergilli

**DOI:** 10.3390/toxins14050340

**Published:** 2022-05-13

**Authors:** Geromy G. Moore, Matthew D. Lebar, Carol H. Carter-Wientjes

**Affiliations:** Southern Regional Research Center, USDA-ARS, New Orleans, LA 70124, USA; matthew.lebar@usda.gov (M.D.L.); carol.carter@usda.gov (C.H.C.-W.)

**Keywords:** VOCs, biocontrol mechanism, aflatoxins, cyclopiazonic acid, indole diterpenes

## Abstract

Previously, authors reported that individual volatile organic compounds (VOCs) emitted by non-aflatoxigenic *Aspergillus flavus* could act as a mechanism of biocontrol to significantly reduce aflatoxins and cyclopiazonic acid (CPA) produced by toxigenic strains. In this study, various combinations and volumes of three mycotoxin-reductive VOCs (2,3-dihydrofuran, 3-octanone and decane) were assessed for their cumulative impacts on four *Aspergillus* strains (LA1–LA4), which were then analyzed for changes in growth, as well as the production of mycotoxins, including aflatoxins, CPA and multiple indole diterpenes. Fungal growth remained minimally inhibited when exposed to various combinations of VOCs. No single combination was able to consistently, or completely, inhibit aflatoxin or CPA across all toxigenic strains tested. However, the combination of 2,3-dihydrofuran and 3-octanone offered the greatest overall reductions in aflatoxin and CPA production. Despite no elimination of their production, findings showed that combining VOCs produced solely by non-aflatoxigenic *A. flavus* still inhibited several agriculturally important mycotoxins, including B and G aflatoxins and CPA. Therefore, other VOC combinations are worth testing as post-harvest biocontrol treatments to ensure the prolonged effectiveness of pre-harvest biocontrol efforts.

## 1. Introduction

Contamination of important food commodities by fungi that produce a multitude of toxic secondary metabolites is a global problem for the economics of agriculture. In some parts of the world, particularly in low to middle income countries, contamination poses serious risks for human and animal health, such as aflatoxicosis (liver toxicity), hepatocarcinoma and multiple types of neurological disruptions [1,2,3]. Compounding the issue of contamination are climate extremes (e.g., extreme heat and drought) that increase crop susceptibility to infection by fungal pathogens [4]. *Aspergillus flavus* and closely related species from the genus’ section *Flavi* are some of the more serious fungal pathogens impacting global economics and health [5,6] due to their ability to infect staple food crops and produce harmful mycotoxins, such as aflatoxin. Therefore, researchers around the globe are developing strategies to prevent these toxin-producing fungi from infecting crop hosts and contaminating them with mycotoxins [7,8].

The use of non-aflatoxigenic *A. flavus* strains as biocontrol is an effective pre-harvest strategy that is implemented worldwide [8,9,10] and offers some post-harvest control under the right conditions [11]. Several mechanisms of action employed by these biocontrol strains have been proposed in the literature, such as competitive exclusion [12], nutrient sequestration [13] and touch inhibition [14]. Our lab has been exploring the mechanism of chemosensing, whereby metabolites secreted by non-aflatoxigenic (biocontrol) *A. flavus* strains trigger a response from mycotoxigenic *Aspergillus* strains to significantly reduce or halt production of major mycotoxins, aflatoxin B1 (AFB_1_) and cyclopiazonic acid (CPA) in particular. One previous study from this lab showed that exposure of each of the three aflatoxin- and CPA-producing *Aspergillus* strains (LA2–LA4) to media infused with uncharacterized extrolites from a non-aflatoxigenic *A. flavus* strain from Louisiana (LA1) resulted in reduced production of both mycotoxins [15]. More recently, our study exposing LA2–LA4 to volatile organic compounds (VOCs) unique to non-aflatoxigenic *A. flavus* showed that several VOCs (administered individually and at different volumes) were also successful at significantly reducing the production of one or more mycotoxins, including AFB_1_, AFG_1_ and CPA [16]. These studies offered evidence of mycotoxin reduction and/or inhibition without the mechanisms of competitive exclusion, nutrient sequestration or touch inhibition. In fact, growth of the mycotoxigenic strains was found to be minimally impacted (i.e., reduced) in our previous chemosensing studies.

In this study, we continued our VOC work by investigating the potential for cumulative effects of the three most impactful VOCs (2,3-dihydrofuran, 3-octanone and decane) from Moore et al. [16] on growth and mycotoxin production. We tested various combinations of these VOCs (cVOCs) to see if there would be enhanced inhibition of AFB_1_ production while simultaneously inhibiting CPA production. We also investigated the production of additional toxic secondary metabolites: AFB_2_, AFG_2_ and indole diterpenes (IDTs), which included aflatrem, aflavazole and aflavinine. According to Coppock and Dziwenka [17], the toxicity levels for AFB_2_ and AFG_2_, respectively, are successively lower than those for AFB_1_ (most potent) and AFG_1_; however, they are still considered major mycotoxins. Aflatrem is a potent tremorgen that has been shown to induce neurological disorders in mice [18,19], so there is potential for this compound to negatively impact humans and animals if consumed. Aflavazole [20] and aflavinine [21,22] are reported to have anti-insectan properties due to their impacts on fungivorous insects, but to our knowledge, their impacts on humans and animals have not been published.

## 2. Results

### 2.1. Impacts of Non-Aflatoxigenic VOC Combinations on Fungal Growth

As observed during our previous extrolite and individual VOC studies, fungal growth for LA1–LA4 was generally unaffected by our cVOCs, with most growth changes lacking statistical support (Tables S1–S5). Though the changes were minor, in many instances different volumes of the same cVOC produced small increases in growth at one volume and small decreases in growth with another volume. The changes in growth were not predictable or volume dependent. Only half of the treatments decreased LA1 growth, with its greatest decrease being 10% from exposure to 20 µL of cVOC 2.3 (Tables S1 and S2). LA2 exhibited more growth increases than decreases from exposure to the different cVOCs. The combination 2.D at the 20 µL volume caused an increase of 13% from the control (Table S1), thereby attaining statistical significance (Table S3; *p* ≤ 0.0309). Conversely, its greatest decrease in growth (13%) was observed with 20 µL of cVOC 2.3. Although minimal, more of the cVOC treatments decreased growth for LA3. Statistically significant differences in LA3 growth were observed with a comparison of the 5 µL vs. 20 µL treatments of cVOC 3.D (Table S4), whereby the 5 µL volume resulted in a 7% decrease, and the 20 µL volume resulted in a 6% increase (*p* ≤ 0.0390). The LA4 strain showed the greatest amount of overall growth increase (up to 18%) from all but one cVOC (Table S1). All volumes of cVOC 3.D increased LA4 growth by 17–18% compared to the control, which also resulted in statistical significance (Table S5; *p* ≤ 0.0388).

**Figure 1:**
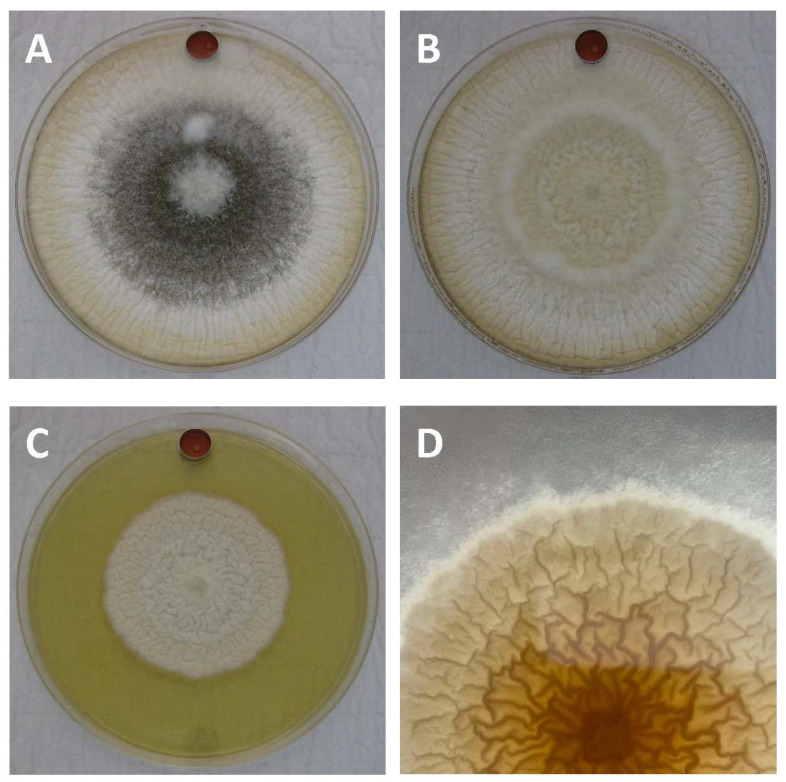
Observed morphological differences for replicates of LA3 with 7 d exposure to 20 µL of combination cVOC 2.3. Replicate (**A**) showed typical colony growth and sclerotial morphology. Replicate (**B**) showed typical growth but lacked sclerotia. Replicate (**C**) showed atypical growth and lacked sclerotia. (**D**) Halo of starved hyphae for replicate C that started at day 4 of incubation.

Occasionally, one or two of the replicates exhibited obvious growth changes like the natural variants observed in our previous VOC study [16]. For example, halos of sparse LA3 hyphae exposed to cVOC 2.3 (2,3-dihydrofuran + 3-octanone) would continue to grow (slowly) after four days of normal growth (Figure 1). These halos were observed for one or two replicates of all strains, except LA4. Whether or not these morphological changes were a direct result of exposure to cVOC 2.3 is unclear, but it is possible that these naturally occurring (rare) variants were more susceptible to this VOC combination.

### 2.2. Assessment of Toxic Secondary Metabolite Production in LA1–LA4 Control Cultures

We assessed the concentrations of aflatoxins, CPA and total IDTs for control cultures of each strain (Table 1). We found that although LA1 did not produce aflatoxin or CPA (as expected), it did produce very high concentrations of the IDTs aflatrem, aflavazole and aflavinine. LA3 also produced IDTs in high concentrations, comparable to those quantities observed in LA1, while LA2 and LA4 produced relatively low levels of IDTs. Regarding aflatoxins, LA4 was the most highly aflatoxigenic strain examined, producing more than three times the amount of B aflatoxins as LA2 and LA3, while LA3 produced more than three times as much CPA as LA2 and LA4.

### 2.3. Impact of Non-Aflatoxigenic VOC Combinations on LA1 Mycotoxin Production

**Figure 2:**
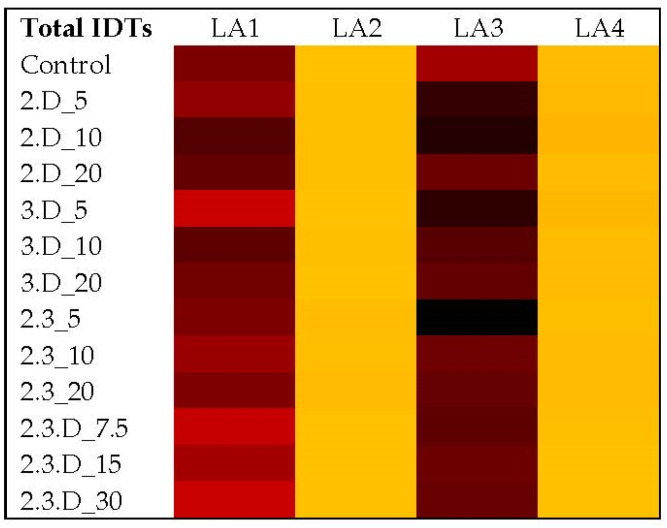
Heatmap showing ppb changes in average levels of combined IDTs (indole diterpenes) from strains LA1–LA4 upon exposure to different combinations and volumes of VOCs. Lighter (yellow) shades indicated low toxin concentrations and became darker (to black) as toxin concentrations increased (166–112,499 ppb). Control = toxins detected when no cVOC was used. For the cVOC codes, 2 = 2,3-dihydrofuran, 3 = 3-octanone, D = decane, and total volumes for each treatment were in µL.

LA1 does not have the genes to produce aflatoxins or CPA, so as expected, neither toxin was detected when the fungus was exposed to various combinations of VOCs. LA1 does, however, produce the IDTs aflatrem, aflavazole and aflavinine (Figure 2; Tables S6 and S11). Total IDT production for LA1 was most reduced from exposure to cVOC 2.3.D at the 7.5 µL volume (47%; *p* = 0.0059) and the 30 µL volume (40%; *p* = 0.0129). None of the other VOC combinations yielded IDT reductions for LA1 greater than 21%. In fact, some cVOC treatments increased the levels of these toxic secondary metabolites (up to 21%).

### 2.4. Impact of Non-Aflatoxigenic VOC Combinations on LA2 Mycotoxin Production

**Figure 3:**
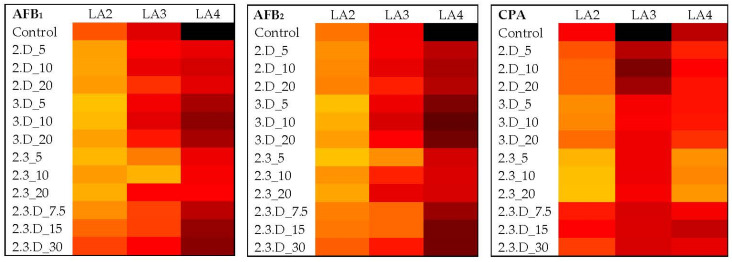
Heatmaps showing changes in average levels of B aflatoxins and CPA from toxic strains LA2–LA4 upon exposure to different combinations and volumes of VOCs. Lighter (yellow) shades indicated low toxin concentrations and became darker (to black) as concentrations increased (AFB_1_: 3308–164,413 ppb; AFB_2_: 40–3471 ppb; CPA: 85–16,651 ppm). For the cVOC codes, 2 = 2,3-dihydrofuran, 3 = 3-octanone, D = decane, and total volumes for each treatment were in µL. Control = toxins detected when no cVOC was used. LA1 does not produce these toxins and was left out of this figure.

Figure 3 illustrates the impacts of each VOC treatment and volume on LA2 aflatoxins and CPA. For AFB_1_, all three volumes of cVOCs 3.D and 2.3 yielded statistically significant reductions ranging from 55% to 84% from control levels (*p* ≤ 0.0448; Tables S7 and S12). However, the most significant reductions were attained with cVOC 2.3 (*p* ≤ 0.0002). The 2.D cVOC significantly reduced AFB_1_ in LA2 by 59% and 60% with the 5 µL and 10 µL treatments, respectively (*p* ≤ 0.0413). The 5 µL volume of cVOC 3.D reduced AFB_2_ levels by 74%, although without statistical support (Tables S8 and S13), and all volumes of cVOC 3.D reduced CPA by 74% (*p* ≤ 0.0010; Tables S9 and S14). The 5 µL volume of cVOC 2.3 decreased AFB_2_ by 75% (*p* < 0.0001), while all volumes of this cVOC reduced CPA by a range of 88–93% (*p* < 0.0001). AFB_2_ reductions by cVOC 2.D were no greater than 27%, while reductions in CPA ranged from 53% to 59% for all volumes (*p* ≤ 0.0133). Combination 2.3.D did not significantly decrease AFB_1_ or AFB_2_ production in LA2, and this cVOC was only moderately effective against CPA production. Total IDT levels for LA2 were low compared to LA1 and were not significantly affected by any treatment (Figure 2; Tables S6 and S15).

### 2.5. Impact of Non-Aflatoxigenic VOC Combinations on LA3 Mycotoxin Production

Despite its inhibitory impacts on LA2 aflatoxins, cVOC 3.D was not an effective treatment for LA3 aflatoxins (Figure 3; Tables S7 and S8). For this strain, cVOC 2.3 was the most effective against AFB_1_, with 73% and 89% decreases from the 5 µL and 10 µL volumes, respectively (*p* ≤ 0.0004; Tables S7 and S16). This VOC combination also decreased AFB_2_ by 75% at the 5 µL volume (*p* = 0.0038; Tables S8 and S17). The two lower volumes of cVOC 2.3.D (7.5 µL and 15 µL) reduced AFB_1_ by 55% (*p* = 0.0003) and AFB_2_ by 63% (*p* ≤ 0.0022). Treatment with 20 µL of cVOC 2.D was (at most) able to reduce AFB_1_ by 50% (*p* = 0.0265). With all volumes, cVOC 2.3 significantly decreased CPA levels in LA3 by 87–91% (*p* < 0.0001; Tables S9 and S18), and cVOC 2.3.D reduced them by 79–80% (*p* < 0.0001). Although cVOCs 2.D and 3.D caused only moderate decreases in LA3’s production of AFB_1_ and AFB_2_, they both significantly reduced its production of CPA by 59–67% (*p* ≤ 0.0288) and 88–93% (*p* < 0.0001), respectively, at all volumes tested. Total IDT levels in LA3 were high, with every VOC combination and treatment volume causing level increases and mostly lacking significance (Tables S6 and S19).

### 2.6. Impact of Non-Aflatoxigenic VOC Combinations on LA4 Mycotoxin Production

**Figure 4:**
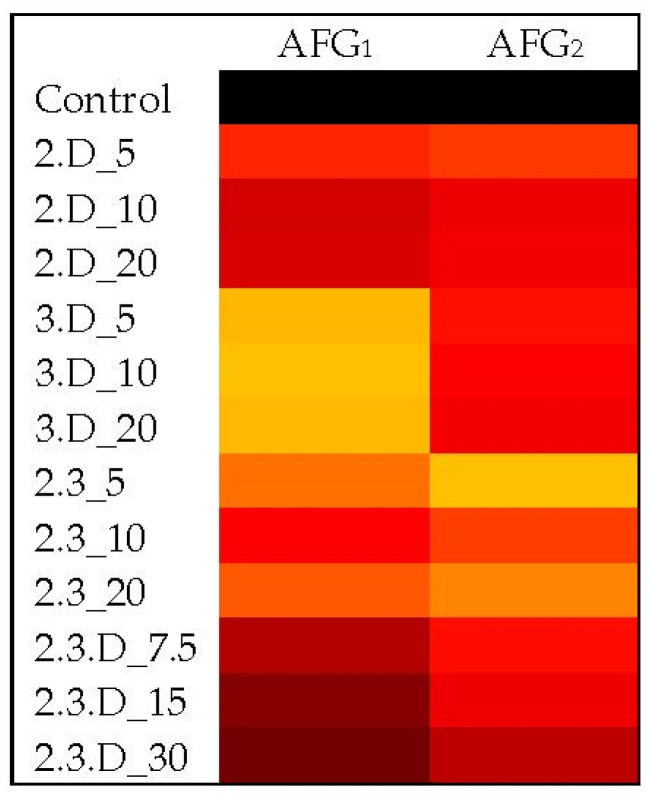
Heatmaps showing changes in average G aflatoxin levels from LA4 upon exposure to different combinations and volumes of VOCs. Lighter (yellow) shades indicated low toxin concentrations and became darker (to black) as concentrations increased (AFG_1_: 20,896–109,084 ppb; AFG_2_: 556–2062 ppb). For the cVOC codes, 2 = 2,3-dihydrofuran, 3 = 3-octanone, D = decane, and total volumes for each treatment were in µL. Control = toxins detected when no cVOC was used. LA1-LA3 do not produce these toxins and were left out of this figure.

For the major LA4 mycotoxins examined, the 3.D, 2.3.D, 2.D and 2.3 cVOC treatments were increasingly effective against AFB_1_ (Figure 3). Both the 3.D and 2.3.D cVOCs were able to reduce AFB_1_ production by an average of 42% (*p* ≤ 0.0420), while cVOC 2.D reduced AFB_1_ by an average of 67% at all volumes (*p* ≤ 0.0006) (Tables S7 and S20). Combination 2.3 decreased production of AFB_1_ by at least 72% (*p* < 0.0001) and AFB_2_ levels by at least 74% (*p* ≤ 0.0022) at all volumes tested (Tables S8 and S21). An AFB_2_ reduction range of 59–65% was attained with LA4 exposure to cVOC 2.D (*p* ≤ 0.0308), followed by cVOC 2.3.D with a range of 41–54% (*p* ≤ 0.0407). AFG_1_ levels were significantly reduced with exposure to nearly all treatments and volumes, with cVOC 3.D being the most impactful by causing reductions at all volumes of around 80% (*p* < 0.0003; Figure 4; Tables S10 and S22). Treatment with all volumes of cVOC 2.3 offered significant AFG_1_ decreases ranging from 71% to 77% (*p* ≤ 0.0001), and all volumes of 2.D decreased this toxin by as much as 73% (*p* ≤ 0.0220). The 2.3.D cVOC at best offered a 50% reduction in AFG_1_, although all volumes had reductions with statistical support (*p* ≤ 0.0407). AFG_2_ levels were reduced by 60–73% with cVOC 2.3 (*p* ≤ 0.0005; Figure 4; Tables S10 and S23). Combination 3.D at all volumes reduced this toxin by more than 50% (*p* ≤ 0.0477). The lowest volumes of cVOCs 2.D (5 µL) and 2.3.D (7.5 µL) reduced AFG_2_ levels by 60% (*p* = 0.0381) and 55% (*p* ≤ 0.0368), respectively. The cVOC 2.3 decreased CPA levels in LA4 by 93–95% (*p* < 0.0001; Tables S9 and S24). Both the cVOCs 2.D and 3.D significantly reduced overall CPA levels by at least 82% (*p* < 0.0001). Treatment with cVOC 2.3.D at the 7.5 µL and 30 µL volumes resulted in CPA decreases of 71% and 53%, respectively (*p* ≤ 0.0216). Like LA2, LA4 had relatively low levels of IDTs and was not significantly affected by any VOC combination (Table S25).

## 3. Discussion

The novelty of this study is based on the importance of *A. flavus* as pre-harvest biocontrol. The global popularity of this mitigation strategy stems from the fact that it is effective at reducing aflatoxin levels in the field, despite the exact mechanism of its effectiveness remaining unclear. None of the non-aflatoxigenic cVOCs we used as treatments in this study caused substantial reductions in growth of the examined strains. At most, growth was reduced by 16% for all four strains. In fact, there were some instances where growth increased by 10% or more. These observations were not surprising, as the individual VOCs used in our previous study had little to no reductive impact on growth and, in some cases, they increased it [16]. Therefore, if the mechanism of an *A. flavus* biocontrol strain involves inhibiting growth of neighboring, aflatoxin-producing strains, then it likely does not use any of the tested VOC compounds to achieve this inhibition. Others have shown significant impacts on mycelial growth, sporulation, conidial germination and expression of aflatoxin biosynthesis genes in *A. flavus* with bacterium-derived VOCs [23]. Reductions in mycelial growth and toxin production have also been reported using yeast-derived VOCs [24,25]. Much of the toxin reduction in these studies is likely due to growth inhibition. Since we observed little to no growth reduction but did observe significant toxin reductions, the VOCs in this study may be directly inhibiting the toxin biosynthesis proteins or interfering with transcription factors regulating those biosynthesis genes.

LA1 has been utilized in our previous studies as a potential biocontrol strain because it does not produce aflatoxins or CPA and is effective in reducing concentrations of these mycotoxins in other strains. Due to our discovery that it produces high amounts of IDTs, LA1 may not be safe for use as a biopesticide on food and feed crops. Whether or not the levels of these IDT mycotoxins are of concern to humans is unclear since metabolites such as aflatrem are currently not regulated like aflatoxins [26]. A feeding study involving mice from the 1980s suggested that a single dose of 3 mg per kg (3000 ppb) of aflatrem caused neurologic deterioration in mice over a matter of weeks [19]. LA1 and LA3 controls produced no less than 10,000 ppb of aflatrem, and even after cVOC treatments, the levels never dropped below 8200 ppb. IDTs are concentrated within sclerotia but can be detected in mycelia in lesser amounts [27], so the high levels of these toxic IDT metabolites in heavily sclerotial strains, such as LA1 and LA3, was not surprising. Since the impact of individual VOCs on total IDT production was not assessed in our previous study, it is unknown if there would have been decreases observed for these strains with individual VOC treatment. A biocontrol strain that produces IDTs is likely more protected from fungivory and would experience maintained presence in a field; however, the potential threats (either synergistic or cumulative) of these metabolites to humans and animals should first be determined before being used to treat food and feed crops. As an alternative, spray or fumigation treatments with the inhibitive compounds produced by non-aflatoxigenic/biocontrol strains, in lieu of the strains themselves, would abate concerns of minor mycotoxins contaminating foods and feeds, as well as other limitations associated with release of live organisms [28].

When comparing major mycotoxins (e.g., AFB_1_ and CPA) in this VOC study to our previous VOC study [16], the individual VOC that had the greatest reductive impact on LA2’s AFB_1_ was decane, which reduced production of this serious mycotoxin by 92% [16]. The most effective cVOC, which involved decane, 3.D, reduced AFB_1_ production in this highly toxic strain by 84% (5 µL volume), so it was less effective but still substantial. No combination pairing a second or third VOC with decane was as effective as decane alone, but treatment of LA2 with cVOC 3.D still yielded a statistically significant reduction in AFB_1_. Regarding the additional mycotoxins examined in this study, VOC combination 3.D continued to be the better treatment against AFB_2_. LA2 does produce sclerotia, which are of the L morphotype, but they were not readily produced in the control or cVOC treatments. The relatively low levels of the three IDTs were not surprising considering no sclerotia were observed. However, this does suggest the detected IDTs observed were inherently derived from LA2 mycelia.

LA3’s AFB_1_ levels were also greatly reduced by decane alone (95%) [16], but the cVOCs involving decane offered no greater than 55% reduction. The combination that offered the greatest reduction in LA3’s AFB_1_ and AFB_2_ levels was cVOC 2.3 (up to 89%). As effective as 2,3-dihydrofuran was at individually reducing AFB_1_, it significantly increased CPA levels for LA3, while 3-octanone on its own was at best marginally effective at reducing CPA in this strain [16]. However, combining these two VOCs significantly decreased CPA by ≥87%. Decane on its own completely inhibited CPA production in LA3 [16]. In this study, however, no combination that included decane was able to completely abolish CPA production, which may be due to the use of a significantly more sensitive instrument used to detect CPA in this study. The previous LC-MS may have missed lower levels of CPA being produced. Exposure to cVOC 2.3.D achieved CPA inhibition levels of about 80%, so perhaps the use of 2,3-dihydrofuran and/or 3-octanone had antagonistic effects on the ability of decane to prevent CPA production. CPA is not currently a regulated mycotoxin but limiting its intake has been recommended [29], so inhibiting its production would be preferable. Total IDT production in this strain was not significantly different when treated with cVOCs. LA3 is an S morphotype strain that produces copious amounts of sclerotia, so the observed high IDT levels in all samples were expected.

LA4 was most sensitive to the three VOCs when tested individually [16]. A combination of the two most impactful VOCs (cVOC 2.D) afforded similar large reductions in production of all examined mycotoxins for this strain. Although not as effective as when used individually, many of these reductions were still substantial and statistically significant. Despite 2,3-dihydrofuran alone completely inhibiting CPA production in LA4 [16], when used in combination with another VOC it failed to maintain complete inhibition of this mycotoxin for LA4. As mentioned above, the previous loss of CPA production may have related to older LC-MS instrumentation. Like the observed changes in total IDT production for LA2, these toxic metabolites in LA4 were little reduced by the presence of any cVOC. The sclerotial morphotype of LA4 was not assessed, and sclerotia were not observed during this study. Therefore, the relatively low IDT levels for this strain are not surprising and likely derive from their inherent presence in mycelia of this strain.

In reference to mycotoxin production, various combinations of these three specific VOCs offered no greater amount of inhibition than when used individually. This was unexpected, given the potential of each compound to reduce aflatoxins and CPA. In nature, fungi tend to produce an assemblage of VOCs, as they respond to their environment [30]. Our forcing together of specific compounds that may not be produced concomitantly could result in limited ability to inhibit mycotoxins, and/or the specific concentrations of each compound we used may have been unnaturally proportioned, thereby limiting the amount of inhibition. Further studies could investigate these and other non-aflatoxigenic VOCs at variable amounts (e.g., 75% + 25% for each two-VOC combination) instead of only equal amounts (e.g., 50% + 50%).

## 4. Conclusions

Overall, potential decreases of greater than 69% in the production of aflatoxins and CPA by cVOC 2.3 indicate this combination could work well as a treatment to inhibit the overall production of mycotoxins. Despite observing some substantial and statistically significant reductions, combining VOCs did not guarantee improved inhibition, since some of the VOCs (tested individually) seemed more effective at reducing aflatoxin and CPA levels. It depends on priority. Is it most important to obtain the greatest reduction in aflatoxin alone and not worry about other mycotoxins? Would it be better to achieve a blanket reduction in multiple mycotoxins greater than 50%? How important are IDTs? Studies are underway to determine if the three VOCs we examined are also produced by LA1 or by commercially available biocontrol strains, such as AF36 and/or NRRL 21882. It is unknown if there is a more effective (i.e., inhibitory) *A. flavus* VOC that has yet to be isolated and/or tested. Moreover, we do not know if there is a specific amount of one or more VOCs that must be attained to completely inhibit production of at least the major mycotoxins. It is also unknown if any unintended chemical reactions occur when combining VOCs, which may render them less effective. These are avenues that necessitate further exploration as we develop research involving chemosensing as a mechanism of *A. flavus* biocontrol.

## 5. Materials and Methods

### 5.1. Louisiana Strains Used in VOC Study

The four Louisiana *Aspergillus* strains used in both of our previous chemosensing studies were first isolated and characterized by Dr. Rebecca Sweany at Louisiana State University [31]. LA1 (KD17, 07-S-3-1-6, SRRC 1588) is a non-aflatoxigenic and non-CPA-producing *A. flavus* strain that forms a mixture of L-type and S-type sclerotia. LA2 (Tox4, 07-C-1-1-1, SRRC 573) is a toxigenic *A. flavus* L-type strain, and LA3 (07-S-2-1-2, SRRC 587) is a toxigenic *A. flavus* S-type strain, both of which produce high levels of B aflatoxins and CPA. LA4 (07-M-S-1-1-1, SRRC 594) is a B+G aflatoxin and CPA-producing species found to share the most recent common ancestor with *A. novoparasiticus* [16].

### 5.2. Experimental Protocol for VOC Study

Experimental setup and mycotoxin extractions were performed as described previously [16] with the following modifications: the lowest volume was 2.5 µL of each VOC, the mid-range volume was 5 µL of each VOC, and the greatest volume was 10 µL of each VOC. Therefore, plates treated with two VOCs still had combined volumes of 5 µL, 10 µL and 20 µL, while plates containing all three VOCs had combined volumes of 7.5 µL, 15 µL and 30 µL. The combinations tested were 2,3-dihydrofuran + decane (2.D), 3-octanone + decane (3.D), 2,3-dihydrofuran + 3-octanone (2.3) and 2,3-dihydrofuran + 3-octanone + decane (2.3.D). As reported in Moore et al. [16], a replicate would sometimes exhibit growth and, subsequently, a toxin profile that was vastly different from the other replicates, which could not be attributed directly to VOC impact. These natural variants (i.e., minor-copy genomes) had potential to cause inconsistencies in the data and inflate standard deviations.

### 5.3. Mycotoxin Analysis

Analyses of aflatoxins (AFB_1_, AFB_2_, AFG_1_ and AFG_2_) were conducted on a Waters Acquity UPLC system (BEH C18 1.7 µm, 2.1 × 50 mm^2^ column) using fluorescence detection (Ex = 365 nm, Em = 440 nm) and a gradient solvent system (0.2 mL/min, solvent A: water; solvent B: methanol): 40% B (0–4.0 min), gradient to 100% B (4.0–9.0 min), 100% B (9.0–13.0 min), then column equilibration 40% B (13.1–18.1 min). Samples were diluted 10- to 1000-fold if the aflatoxin signal saturated the detector.

CPA and total IDT analyses were conducted on a Waters Acquity UPLC system coupled with a Waters Xevo G2 XS QTOF mass spectrometer equipped with a Z-spray ionization source running in ESI+ mode using MassLynx 4.2 software with the following QTOF settings: Source temperature: 100 °C, Desolvation temperature: 250 °C, Desolvation gas flow: 600 L/h, Cone gas flow: 50 L/h, Capillary voltage: 3.0 kV, Sampling cone voltage: 40 V. Analyses were performed in sensitivity and continuum mode, with a mass range of m/z 50–1200 and a scan time of 0.1 s. A data-independent acquisition method with elevated collision energy (MSE) was used with 6 eV low energy and a high-energy ramp from 15 to 45 eV. Chromatography was conducted on a Waters BEH C18 1.7 µm, 2.1 × 50 mm^2^ column with the following gradient solvent system (0.5 mL/min, solvent A: 0.1% formic acid in water; solvent B: 0.1% formic acid in acetonitrile): 5% B (0–1.25 min), gradient to 25% B (1.25–1.5 min), gradient to 100% B (1.5–5.0 min), 100% B (5.0–7.5 min), then column equilibration 5% B (7.6–10.1 min). Data were analyzed on Waters UNIFI 1.9.4 software using the “Quantify Assay Tof 2D” analysis method with lock mass corrected by UNIFI.

Standards (Sigma-Aldrich, St. Louis, MO) were used to identify and quantify AFB_1_, AFB_2_, AFG_1_, AFG_2_ and CPA. Aflatoxin concentrations were expressed in ng aflatoxin per g agar (ppb). CPA concentrations were expressed in µg CPA per g agar (ppm). Aflatrem, aflavazole and aflavinine standards were kindly provided by Dr. James Gloer, University of Iowa, United States. The observed concentrations of these three IDTs were combined and averaged and are reported in ng of total IDTs per g agar (ppb).

### 5.4. Handling of Data

Standard deviation from the mean was determined for each set of three replicates, using STDEV.S in Microsoft Excel. Statistical significance was assessed between control and experimental values, as well as among treatment volumes for each VOC combination by two-way ANOVA followed by Tukey’s multiple comparisons test, using GraphPad Prism (GraphPad Software, Inc., San Diego, CA, USA).

## Figures and Tables

**Table 1:** Average control values for examined mycotoxins produced by strains LA1–LA4.

Strain	AFB_1_ (ppb)	AFB_2_ (ppb)	AFG_1_ (ppb)	AFG_2_ (ppb)	CPA (ppm)	Total IDTs (ppb)
LA1	0	0	0	0	0	81,538 (7785)
LA2	20,975 (3183)	163 (20)	0	0	1202 (180)	280 (164)
LA3	54,084 (5384)	492 (33)	0	0	16,651 (32)	65,757 (32,632)
LA4	164,413 (7179)	3471 (800)	109,084 (13,413)	2062 (316)	5157 (400)	605 (351)

Numbers in parentheses are standard deviations from the mean.

## Supplementary Materials

The following supporting information can be downloaded at: www.mdpi.com/article/10.3390/toxins14050340/s1, Table S1: Average growth (mm) assessed for each LA Strain and VOC treatment; Table S2: Results of two-way ANOVA and Tukey’s test comparing LA1 control growth with each VOC combination treatment; Table S3: Results of two-way ANOVA and Tukey’s test comparing LA2 control growth with each VOC combination treatment; Table S4: Results of two-way ANOVA and Tukey’s test comparing LA3 control growth with each VOC combination treatment; Table S5: Results of two-way ANOVA and Tukey’s test comparing LA4 control growth with each VOC combination treatment; Table S6: Average total IDT concentrations assessed for each LA Strain and VOC treatment; Table S7: Average AFB_1_ concentrations assessed for toxic LA strains and VOC treatments; Table S8: Average AFB_2_ concentrations assessed for toxic LA strains and VOC treatments; Table S9: Average CPA concentrations assessed for toxic LA strains and VOC treatments; Table S10: Average AFG concentrations assessed for LA4 with each VOC treatment; Table S11: Results of two-way ANOVA and Tukey’s test comparing LA1 control IDTs with each VOC combination treatment; Table S12: Results of two-way ANOVA and Tukey’s test comparing LA2 control AFB_1_ with each VOC combination treatment; Table S13: Results of two-way ANOVA and Tukey’s test comparing LA2 control AFB_2_ with each VOC combination treatment; Table S14: Results of two-way ANOVA and Tukey’s test comparing LA2 control CPA with each VOC combination treatment; Table S15: Results of two-way ANOVA and Tukey’s test comparing LA2 control IDTs with each VOC combination treatment; Table S16: Results of two-way ANOVA and Tukey’s test comparing LA3 control AFB_1_ with each VOC combination treatment; Table S17: Results of two-way ANOVA and Tukey’s test comparing LA3 control AFB_2_ with each VOC combination treatment; Table S18: Results of two-way ANOVA and Tukey’s test comparing LA3 control CPA with each VOC combination treatment; Table S19: Results of two-way ANOVA and Tukey’s test comparing LA3 control IDTs with each VOC combination treatment; Table S20: Results of two-way ANOVA and Tukey’s test comparing LA4 control AFB_1_ with each VOC combination treatment; Table S21: Results of two-way ANOVA and Tukey’s test comparing LA4 control AFB_2_ with each VOC combination treatment; Table S22: Results of two-way ANOVA and Tukey’s test comparing LA4 control AFG_1_ with each VOC combination treatment; Table S23: Results of two-way ANOVA and Tukey’s test comparing LA4 control AFG_2_ with each VOC combination treatment; Table S24: Results of two-way ANOVA and Tukey’s test comparing LA4 control CPA with each VOC combination treatment; Table S25: Results of two-way ANOVA and Tukey’s test comparing LA4 control IDTs with each VOC combination treatment.
