# Supplementary material for: Cumulative Effects of Non-Aflatoxigenic Aspergillus flavus Volatile Organic Compounds to Abate Toxin Production by Mycotoxigenic Aspergilli
Source: Toxins (Basel). 2022 May 13;14(5):340. doi: 10.3390/toxins14050340 (PMC9148032; doi:10.3390/toxins14050340)
Supplement: Supplementary file 1 [file toxins-14-00340-s001.zip › toxins-1668610-supplementary.pdf]

Supplementary Materials: Cumulative Effects of Non-Aflatoxigenic *Aspergillus flavus* Volatile Organic Compounds to Abate Toxin Production by Mycotoxigenic Aspergilli

Table S1. Average growth (mm) assessed for each LA Strain and VOC treatment.

**Treatment[1]**	**Growth[2]**	**Percent Change[3]**
	**LA1 (SRRC 1588)**	
Control	80.2 (1)	N/A
3.D_5	80.6 (5)	<1% inc.
3.D_10	79.3 (3.4)	1% dec.
3.D_20	83.9 (4.7)	5% inc.
2.D_5	80.6 (4)	<1% inc.
2.D_10	79.4 (4.4)	1% dec.
2.D_20	81.5 (6.4)	2% inc.
2.3_5	87.9 (0.3)	9% inc.
2.3_10	80.5 (4.6)	<1% inc.
2.3_20	72.2 (13.6)	10% dec.
2.3.D_7.5	79.3 (4.6)	1% dec.
2.3.D_15	73.9 (6.5)	8% dec.
2.3.D_30	78.7 (5.6)	2% dec.
	**LA2 (SRRC 573)**	
Control	78.6 (1.6)	N/A
3.D_5	87.3 (4.6)	11% inc.
3.D_10	87.8 (3.2)	12% inc.
3.D_20	86.1 (2.7)	10% inc.
2.D_5	87.8 (4.8)	12% inc.
2.D_10	83 (2.3)	6% inc.
2.D_20	89 (2.1)	13% inc.
2.3_5	83.9 (0.5)	6% inc.
2.3_10	74.4 (11.3)	5% dec.
2.3_20	68.1 (13.1)	13% dec.
2.3.D_15	77.7 (0.1)	1% dec.
2.3.D_7.5	77 (1.6)	2% dec.
2.3.D_30	74.8 (3.2)	5% dec.
	**LA3 (SRRC 587)**	
Control	84 (2.6)	N/A
3.D_5	78.9 (4.3)	7% dec.
3.D_10	86 (2.3)	2% inc.
3.D_20	88.7 (3.6)	6% inc.
2.D_5	83.6 (2.9)	<1% dec.
2.D_10	83.1 (4.2)	1% dec.
2.D_20	84.4 (1.6)	<1% inc.
2.3_5	87.1 (0.5)	4% inc.

2.3_10	73.1 (13.9)	13% dec.
2.3_20	70.9 (15)	16% dec.
2.3.D_7.5	80.5 (1)	4% dec.
2.3.D_15	80.4 (0.4)	4% dec.
2.3.D_30	80.9 (0.4)	4% dec.
**LA4 (SRRC 594)**		
Control	75.1 (9.1)	N/A
3.D_5	88.3 (1.6)	17% inc.
3.D_10	88.3 (1.2)	18% inc.
3.D_20	88.9 (0.8)	18% inc.
2.D_5	86.3 (2)	15% inc.
2.D_10	85.2 (3.3)	13% inc.
2.D_20	79.6 (6.8)	6% inc.
2.3_5	84.4 (0.8)	12% inc.
2.3_10	70.6 (10.5)	6% dec.
2.3_20	73 (10.1)	3% dec.
2.3.D_7.5	82.5 (1.2)	10% inc.
2.3.D_15	83.1 (0.9)	11% inc.
2.3.D_30	83.1 (1.6)	11% inc.

[1] VOC combinations (2 = 2,3-dihydrofuran, 3 = 3-octanone, D = decane) and total volumes for each treatment (in µl). Control = growth observed when no VOC combinations were used.

[2] Average colony diameters (in mm) across three replicate plates assessed for each VOC treatment. The number in parentheses is the standard deviation from the mean.

[3] Percent (%) change in growth from the control value. dec. = decrease; inc. = increase. N/A indicates no value could be assessed.

Table S2. Results of two-way ANOVA and Tukey's test comparing LA1 control growth with each VOC combination treatment.

3.D ANOVA	SS (Type III)[a]	DF[b]	MS[c]	F (DFn, DFd)[d]	*P* value
Replicate	14.64	2	7.318	F (2, 6) = 0.4174	0.6765
Treatment	36.50	3	12.17	F (3, 6) = 0.6939	0.5886
**3.D Tukey's**	**Mean Diff.[e]**	**95.00% CI of diff.[f]**	**Below threshold?**	**Summary[g]**	**Adjusted *P* Value**
Control vs. 3.D_5	-0.3833	-12.22 to 11.45	No	ns	0.9994
Control vs. 3.D_10	0.9267	-10.91 to 12.76	No	ns	0.9923
Control vs. 3.D_20	-3.693	-15.53 to 8.141	No	ns	0.7131
3.D_5 vs. 3.D_10	1.310	-10.52 to 13.14	No	ns	0.9791
3.D_5 vs. 3.D_20	-3.310	-15.14 to 8.525	No	ns	0.7717
3.D_10 vs. 3.D_20	-4.620	-16.45 to 7.215	No	ns	0.5677
**2.D ANOVA**	**SS (Type III)[a]**	**DF[b]**	**MS[c]**	**F (DFn, DFd)[d]**	***P* value**
Replicate	87.88	2	43.94	F (2, 6) = 4.039	0.0774
Treatment	6.784	3	2.261	F (3, 6) = 0.2079	0.8874
**2.D Tukey's**	**Mean Diff.[e]**	**95.00% CI of diff.[f]**	**Below threshold?**	**Summary[g]**	**Adjusted *P* Value**
Control vs. 2.D_5	-0.3567	-9.679 to 8.966	No	ns	0.9991
Control vs. 2.D_10	0.7867	-8.536 to 10.11	No	ns	0.9904
Control vs. 2.D_20	-1.307	-10.63 to 8.016	No	ns	0.9596
2.D_5 vs. 2.D_10	1.143	-8.179 to 10.47	No	ns	0.9721
2.D_5 vs. 2.D_20	-0.9500	-10.27 to 8.373	No	ns	0.9835
2.D_10 vs. 2.D_20	-2.093	-11.42 to 7.229	No	ns	0.8621
**2.3 ANOVA**	**SS (Type III)[a]**	**DF[b]**	**MS[c]**	**F (DFn, DFd)[d]**	***P* value**
Replicate	2.491	2	1.245	F (2, 6) = 0.09151	0.9138
Treatment	235.3	3	78.44	F (3, 6) = 5.764	0.0336
**2.3 Tukey's**	**Mean Diff.[e]**	**95.00% CI of diff.[f]**	**Below threshold?**	**Summary[g]**	**Adjusted *P* Value**
Control vs. 2.3_5	-7.687	-18.11 to 2.740	No	ns	0.1476
Control vs. 2.3_10	-0.3100	-10.74 to 10.12	No	ns	0.9996
Control vs. 2.3_20	4.690	-5.737 to 15.12	No	ns	0.4643
2.3_5 vs. 2.3_10	7.377	-3.050 to 17.80	No	ns	0.1671
2.3_5 vs. 2.3_20	12.38	1.950 to 22.80	Yes	*	0.0243
2.3_10 vs. 2.3_20	5.000	-5.427 to 15.43	No	ns	0.4168
**2.3.D ANOVA**	**SS (Type III)[a]**	**DF[b]**	**MS[c]**	**F (DFn, DFd)[d]**	***P* value**
Replicate	0.6254	2	0.3127	F (2, 6) = 0.009870	0.9902
Treatment	71.02	3	23.67	F (3, 6) = 0.7471	0.5623
**2.3.D Tukey's**	**Mean Diff.[e]**	**95.00% CI of diff.[f]**	**Below threshold?**	**Summary[g]**	**Adjusted *P* Value**
Control vs. 2.3.D_7.5	0.8533	-15.06 to 16.76	No	ns	0.9975
Control vs. 2.3.D_15	6.260	-9.650 to 22.17	No	ns	0.5621
Control vs. 2.3.D_30	1.460	-14.45 to 17.37	No	ns	0.9878
2.3.D_7.5 vs. 2.3.D_15	5.407	-10.50 to 21.32	No	ns	0.6614
2.3.D_7.5 vs. 2.3.D_30	0.6067	-15.30 to 16.52	No	ns	0.9991
2.3.D_15 vs. 2.3.D_30	-4.800	-20.71 to 11.11	No	ns	0.7322

[a] Partial (non-sequential) Sum of Squares. [b] Degrees of Freedom [c] Mean Squares [d] F ratios based on Degrees of Freedom. n = numerator, d = denominator [e] Difference from the control mean. Negative values indicate enhanced growth and positive values indicate reduced growth. [f] Confidence interval (CI) of difference from control [g] Not significant (ns) > 0.05, * $P \leq 0.05$, ** $P \leq 0.01$, *** $P \leq 0.001$, **** $P \leq 0.0001$.

Table S3. Results of two-way ANOVA and Tukey's test comparing LA2 control growth with each VOC combination treatment.

**3.D ANOVA**	**SS (Type III)[a]**	**DF[b]**	**MS[c]**	**F (DFn, DFd)[d]**	***P* value**
Replicate	3.164	2	1.582	F (2, 6) = 0.1219	0.8874
Treatment	166.0	3	55.32	F (3, 6) = 4.261	0.0621
**3.D Tukey's**	**Mean Diff.[e]**	**95.00% CI of diff.[f]**	**Below threshold?**	**Summary[g]**	**Adjusted *P* Value**
Control vs. 3.D_5	-8.717	-18.90 to 1.468	No	ns	0.0899
Control vs. 3.D_10	-9.197	-19.38 to 0.9877	No	ns	0.0741
Control vs. 3.D_20	-7.487	-17.67 to 2.698	No	ns	0.1489
3.D_5 vs. 3.D_10	-0.4800	-10.66 to 9.704	No	ns	0.9983
3.D_5 vs. 3.D_20	1.230	-8.954 to 11.41	No	ns	0.9733
3.D_10 vs. 3.D_20	1.710	-8.474 to 11.89	No	ns	0.9342
**2.D ANOVA**	**SS (Type III)[a]**	**DF[b]**	**MS[c]**	**F (DFn, DFd)[d]**	***P* value**
Replicate	5.056	2	2.528	F (2, 6) = 0.2335	0.7986
Treatment	205.2	3	68.39	F (3, 6) = 6.317	0.0275
**2.D Tukey's**	**Mean Diff.[e]**	**95.00% CI of diff.[f]**	**Below threshold?**	**Summary[g]**	**Adjusted *P* Value**
Control vs. 2.D_5	-9.153	-18.45 to 0.1467	No	ns	0.0533
Control vs. 2.D_10	-4.363	-13.66 to 4.937	No	ns	0.4329
Control vs. 2.D_20	-10.45	-19.75 to -1.147	Yes	*	0.0309
2.D_5 vs. 2.D_10	4.790	-4.510 to 14.09	No	ns	0.3646
2.D_5 vs. 2.D_20	-1.293	-10.59 to 8.007	No	ns	0.9605
2.D_10 vs. 2.D_20	-6.083	-15.38 to 3.217	No	ns	0.2086
**2.3 ANOVA**	**SS (Type III)[a]**	**DF[b]**	**MS[c]**	**F (DFn, DFd)[d]**	***P* value**
Replicate	15.92	2	7.959	F (2, 6) = 2.042	0.2106
Treatment	112.7	3	37.57	F (3, 6) = 9.638	0.0104
**2.3 Tukey's**	**Mean Diff.[e]**	**95.00% CI of diff.[f]**	**Below threshold?**	**Summary[g]**	**Adjusted *P* Value**
Control vs. 2.3_5	-5.263	-10.84 to 0.3168	No	ns	0.0628
Control vs. 2.3_10	-0.1633	-5.743 to 5.417	No	ns	0.9996
Control vs. 2.3_20	3.310	-2.270 to 8.890	No	ns	0.2678
2.3_5 vs. 2.3_10	5.100	-0.4801 to 10.68	No	ns	0.0708
2.3_5 vs. 2.3_20	8.573	2.993 to 14.15	Yes	**	0.0072
2.3_10 vs. 2.3_20	3.473	-2.107 to 9.053	No	ns	0.2377
**2.3.D ANOVA**	**SS (Type III)[a]**	**DF[b]**	**MS[c]**	**F (DFn, DFd)[d]**	***P* value**
Replicate	2.929	2	1.465	F (2, 6) = 0.3147	0.7414
Treatment	23.98	3	7.993	F (3, 6) = 1.717	0.2622
**2.3.D Tukey's**	**Mean Diff.[e]**	**95.00% CI of diff.[f]**	**Below threshold?**	**Summary[g]**	**Adjusted *P* Value**
Control vs. 2.3.D_7.5	1.620	-4.478 to 7.718	No	ns	0.7961
Control vs. 2.3.D_15	0.9333	-5.165 to 7.031	No	ns	0.9486
Control vs. 2.3.D_30	3.833	-2.265 to 9.931	No	ns	0.2317
2.3.D_7.5 vs. 2.3.D_15	-0.6867	-6.785 to 5.411	No	ns	0.9781
2.3.D_7.5 vs. 2.3.D_30	2.213	-3.885 to 8.311	No	ns	0.6182
2.3.D_15 vs. 2.3.D_30	2.900	-3.198 to 8.998	No	ns	0.4229

[a] Partial (non-sequential) Sum of Squares [b] Degrees of Freedom [c] Mean Squares [d] F ratios based on Degrees of Freedom. n = numerator, d = denominator [e] Difference from the control mean. Negative values indicate enhanced growth and positive values indicate reduced growth. [f] Confidence interval (CI) of difference from control [g] Not significant (ns) > 0.05, * $P \leq 0.05$, ** $P \leq 0.01$, *** $P \leq 0.001$, **** $P \leq 0.0001$.

Table S4. Results of two-way ANOVA and Tukey's test comparing LA3 control growth with each VOC combination treatment.

3.D ANOVA	SS (Type III)[a]	DF[b]	MS[c]	F (DFn, DFd)[d]	*P* value
Replicate	28.67	2	14.33	F (2, 6) = 1.493	0.2978
Treatment	154.7	3	51.57	F (3, 6) = 5.370	0.0390
**3.D Tukey's**	**Mean Diff.[e]**	**95.00% CI of diff.[f]**	**Below threshold?**	**Summary[g]**	**Adjusted *P* Value**
Control vs. 3.D_5	5.163	-3.595 to 13.92	No	ns	0.2718
Control vs. 3.D_10	-1.977	-10.74 to 6.782	No	ns	0.8605
Control vs. 3.D_20	-4.640	-13.40 to 4.119	No	ns	0.3445
3.D_5 vs. 3.D_10	-7.140	-15.90 to 1.619	No	ns	0.1065
3.D_5 vs. 3.D_20	-9.803	-18.56 to -1.045	Yes	*	0.0313
3.D_10 vs. 3.D_20	-2.663	-11.42 to 6.095	No	ns	0.7278
**2.D ANOVA**	**SS (Type III)[a]**	**DF[b]**	**MS[c]**	**F (DFn, DFd)[d]**	***P* value**
Replicate	28.59	2	14.30	F (2, 6) = 2.001	0.2159
Treatment	2.847	3	0.9490	F (3, 6) = 0.1328	0.9370
**2.D Tukey's**	**Mean Diff.[e]**	**95.00% CI of diff.[f]**	**Below threshold?**	**Summary[g]**	**Adjusted *P* Value**
Control vs. 2.D_5	0.4033	-7.151 to 7.958	No	ns	0.9975
Control vs. 2.D_10	0.9733	-6.581 to 8.528	No	ns	0.9680
Control vs. 2.D_20	-0.3333	-7.888 to 7.221	No	ns	0.9986
2.D_5 vs. 2.D_10	0.5700	-6.985 to 8.125	No	ns	0.9931
2.D_5 vs. 2.D_20	-0.7367	-8.291 to 6.818	No	ns	0.9855
2.D_10 vs. 2.D_20	-1.307	-8.861 to 6.248	No	ns	0.9288
**2.3 ANOVA**	**SS (Type III)[a]**	**DF[b]**	**MS[c]**	**F (DFn, DFd)[d]**	***P* value**
Replicate	22.83	2	11.41	F (2, 6) = 2.541	0.1587
Treatment	141.1	3	47.05	F (3, 6) = 10.47	0.0085
**2.3 Tukey's**	**Mean Diff.[e]**	**95.00% CI of diff.[f]**	**Below threshold?**	**Summary[g]**	**Adjusted *P* Value**
Control vs. 2.3_5	-3.070	-9.061 to 2.921	No	ns	0.3683
Control vs. 2.3_10	4.500	-1.491 to 10.49	No	ns	0.1392
Control vs. 2.3_20	5.383	-0.6077 to 11.37	No	ns	0.0754
2.3_5 vs. 2.3_10	7.570	1.579 to 13.56	Yes	*	0.0183
2.3_5 vs. 2.3_20	8.453	2.462 to 14.44	Yes	*	0.0109
2.3_10 vs. 2.3_20	0.8833	-5.108 to 6.874	No	ns	0.9536
**2.3.D ANOVA**	**SS (Type III)[a]**	**DF[b]**	**MS[c]**	**F (DFn, DFd)[d]**	***P* value**
Replicate	3.821	2	1.911	F (2, 6) = 0.9050	0.4534
Treatment	26.77	3	8.924	F (3, 6) = 4.227	0.0631
**2.3.D Tukey's**	**Mean Diff.[e]**	**95.00% CI of diff.[f]**	**Below threshold?**	**Summary[g]**	**Adjusted *P* Value**
Control vs. 2.3.D_7.5	3.523	-0.5833 to 7.630	No	ns	0.0892
Control vs. 2.3.D_15	3.597	-0.5100 to 7.703	No	ns	0.0828
Control vs. 2.3.D_30	3.167	-0.9400 to 7.273	No	ns	0.1281
2.3.D_7.5 vs. 2.3.D_15	0.07333	-4.033 to 4.180	No	ns	> 0.9999
2.3.D_7.5 vs. 2.3.D_30	-0.3567	-4.463 to 3.750	No	ns	0.9896
2.3.D_15 vs. 2.3.D_30	-0.4300	-4.537 to 3.677	No	ns	0.9822

[a] Partial (non-sequential) Sum of Squares [b] Degrees of Freedom [c] Mean Squares [d] F ratios based on Degrees of Freedom. n = numerator, d = denominator [e] Difference from the control mean. Negative values indicate enhanced growth and positive values indicate reduced growth. [f] Confidence interval (CI) of difference from control [g] Not significant (ns) > 0.05, * $P \leq 0.05$, ** $P \leq 0.01$, *** $P \leq 0.001$, **** $P \leq 0.0001$.

Table S5. Results of two-way ANOVA and Tukey's test comparing LA4 control growth with each VOC combination treatment

3.D ANOVA	SS (Type III)[a]	DF[b]	MS[c]	F (DFn, DFd)[d]	[illegible]
Replicate	59.28	2	29.64	F (2, 6) = 1.545	[illegible]
Treatment	403.1	3	134.4	F (3, 6) = 7.005	[illegible]
**3.D Tukey's**	**Mean Diff.[e]**	**95.00% CI of diff.[f]**	**Below threshold?**	**Summary[g]**	[illegible]
Control vs. 3.D_5	-13.19	-25.57 to -0.8142	Yes	*	[illegible]
Control vs. 3.D_10	-13.17	-25.55 to -0.7942	Yes	*	[illegible]
Control vs. 3.D_20	-13.76	-26.14 to -1.378	Yes	*	[illegible]
3.D_5 vs. 3.D_10	0.02000	-12.36 to 12.40	No	ns	[illegible]
3.D_5 vs. 3.D_20	-0.5633	-12.94 to 11.82	No	ns	[illegible]
3.D_10 vs. 3.D_20	-0.5833	-12.96 to 11.80	No	ns	[illegible]
**2.D ANOVA**	**SS (Type III)[a]**	**DF[b]**	**MS[c]**	**F (DFn, DFd)[d]**	[illegible]
Replicate	37.30	2	18.65	F (2, 6) = 0.4489	[illegible]
Treatment	243.6	3	81.21	F (3, 6) = 1.955	[illegible]
**2.D Tukey's**	**Mean Diff.[e]**	**95.00% CI of diff.[f]**	**Below threshold?**	**Summary[g]**	[illegible]
Control vs. 2.D_5	-11.21	-29.43 to 7.004	No	ns	[illegible]
Control vs. 2.D_10	-10.05	-28.26 to 8.170	No	ns	[illegible]
Control vs. 2.D_20	-4.453	-22.67 to 13.76	No	ns	[illegible]
2.D_5 vs. 2.D_10	1.167	-17.05 to 19.38	No	ns	[illegible]
2.D_5 vs. 2.D_20	6.760	-11.46 to 24.98	No	ns	[illegible]
2.D_10 vs. 2.D_20	5.593	-12.62 to 23.81	No	ns	[illegible]
**2.3 ANOVA**	**SS (Type III)[a]**	**DF[b]**	**MS[c]**	**F (DFn, DFd)[d]**	[illegible]
Replicate	180.8	2	90.40	F (2, 6) = 4.211	[illegible]
Treatment	182.1	3	60.72	F (3, 6) = 2.828	[illegible]
**2.3 Tukey's**	**Mean Diff.[e]**	**95.00% CI of diff.[f]**	**Below threshold?**	**Summary[g]**	[illegible]
Control vs. 2.3_5	-9.310	-22.41 to 3.786	No	ns	[illegible]
Control vs. 2.3_10	-0.5233	-13.62 to 12.57	No	ns	[illegible]
Control vs. 2.3_20	-0.4500	-13.55 to 12.65	No	ns	[illegible]
2.3_5 vs. 2.3_10	8.787	-4.309 to 21.88	No	ns	[illegible]
2.3_5 vs. 2.3_20	8.860	-4.236 to 21.96	No	ns	[illegible]
2.3_10 vs. 2.3_20	0.07333	-13.02 to 13.17	No	ns	[illegible]
**2.3.D ANOVA**	**SS (Type III)[a]**	**DF[b]**	**MS[c]**	**F (DFn, DFd)[d]**	[illegible]
Replicate	27.37	2	13.69	F (2, 6) = 0.5564	[illegible]
Treatment	136.4	3	45.45	F (3, 6) = 1.848	[illegible]
**2.3.D Tukey's**	**Mean Diff.[e]**	**95.00% CI of diff.[f]**	**Below threshold?**	**Summary[g]**	[illegible]
Control vs. 2.3.D_7.5	-7.333	-21.35 to 6.685	No	ns	[illegible]
Control vs. 2.3.D_15	-8.000	-22.02 to 6.018	No	ns	[illegible]
Control vs. 2.3.D_30	-7.950	-21.97 to 6.068	No	ns	[illegible]
2.3.D_7.5 vs. 2.3.D_15	-0.6667	-14.68 to 13.35	No	ns	[illegible]
2.3.D_7.5 vs. 2.3.D_30	-0.6167	-14.63 to 13.40	No	ns	[illegible]
2.3.D_15 vs. 2.3.D_30	0.05000	-13.97 to 14.07	No	ns	[illegible]

[a] Partial (non-sequential) Sum of Squares [b] Degrees of Freedom [c] Mean Squares [d] F ratios based on Degrees of Freedom. n = numerator, d = denominator [e] Difference from the control mean. Negative values indicate enhanced growth and positive values indicate reduced growth. [f] Confidence interval (CI) of difference from control [g] Not significant (ns) > 0.05, * $P \leq 0.05$, ** $P \leq 0.01$, *** $P \leq 0.001$, **** $P \leq 0.0001$.

Table S6. Average total IDT concentrations assessed for each LA Strain and VOC treatment.

Treatment[1]	Concentration (ppb)[2]	Percent Change[3]
	**LA1 (SRRC 1588)**	
Control	81538 (7785)	N/A
3.D_5	64090 (14733)	21% dec.
3.D_10	90842 (14852)	11% inc.
3.D_20	68187 (31895)	16% dec.
2.D_5	72210 (16080)	11% dec.
2.D_10	98556 (26232)	21% inc.
2.D_20	92787 (15376)	14% inc.
2.3_5	81765 (17803)	0.3% inc.
2.3_10	69139 (10111)	15% dec.
2.3_20	79742 (6426)	2% dec.
2.3.D_7.5	43505 (6378)	47% dec.
2.3.D_15	64573 (9715)	21% dec.
2.3.D_30	49177 (6636)	40% dec.
	**LA2 (SRRC 573)**	
Control	280 (164)	N/A
3.D_5	232 (110)	17% dec.
3.D_10	422 (330)	51% inc.
3.D_20	216 (54)	23% dec.
2.D_5	444 (213)	59% inc.
2.D_10	330 (127)	18% inc.
2.D_20	432 (296)	54% inc.
2.3_5	631 (330)	125% inc.
2.3_10	487 (352)	74% inc.
2.3_20	785 (877)	180% inc.
2.3.D_7.5	242 (107)	14% dec.
2.3.D_15	243 (70)	13% dec.
2.3.D_30	166 (54)	41% dec.
	**LA3 (SRRC 587)**	
Control	65757 (32632)	N/A
3.D_5	114526 (7388)	74% inc.
3.D_10	97115 (9715)	48% inc.
3.D_20	92186 (15787)	40% inc.
2.D_5	112499 (6430)	71% inc.
2.D_10	119401 (27346)	82% inc.
2.D_20	88700 (43243)	35% inc.
2.3_5	134137 (17392)	104% inc.
2.3_10	87617 (60387)	33% inc.
2.3_20	91264 (12145)	39% inc.
2.3.D_7.5	94123 (3174)	43% inc.
2.3.D_15	88565 (11750)	35% inc.
2.3.D_30	90477 (4721)	38% inc.
	**LA4 (SRRC 594)**	
Control	605 (351)	N/A

3.D_5	1376 (862)	127% inc.
3.D_10	671 (184)	11% inc.
3.D_20	589 (286)	3% dec.
2.D_5	1273 (600)	110% inc.
2.D_10	1748 (1354)	189% inc.
2.D_20	605 (302)	0.01% dec.
2.3_5	502 (49)	17% dec.
2.3_10	810 (485)	34% inc.
2.3_20	821 (381)	36% inc.
2.3.D_7.5	452 (433)	25% dec.
2.3.D_15	253 (53)	58% dec.
2.3.D_30	207 (18)	66% dec.

[1] VOC combinations (2 = 2,3-dihydrofuran, 3 = 3-octanone, D = decane) and total volumes for each treatment (in μl). Control = toxins detected when no VOC combinations were used.

[2] Average mycotoxin concentrations (in ppb) assessed for each VOC treatment. The number in parentheses is the standard deviation from the mean.

[3] Percent (%) change in mycotoxin production from the control value. dec. = decrease; inc. = increase. N/A indicates no value could be assessed.

Table S7. Average AFB_1 concentrations assessed for toxic LA strains and VOC treatments.

Treatment[1]	Concentration (ppb)[2]	Percent Change[3]
	**LA2 (SRRC 573)**	
Control	20974 (3183)	N/A
3.D_5	3308 (1219)	84% dec.
3.D_10	4570 (1804)	78% dec.
3.D_20	8633 (7789)	59% dec.
2.D_5	8601 (1658)	59% dec.
2.D_10	8350 (1442)	60% dec.
2.D_20	9448 (7495)	55% dec.
2.3_5	5032 (788)	76% dec.
2.3_10	9492 (1746)	55% dec.
2.3_20	6541 (3602)	69% dec.
2.3.D_7.5	11717 (9443)	44% dec.
2.3.D_15	18228 (5649)	13% dec.
2.3.D_30	24182 (1547)	15% inc.
	**LA3 (SRRC 587)**	
Control	54084 (5384)	N/A
3.D_5	41709 (7730)	23% dec.
3.D_10	52719 (23457)	3% dec.
3.D_20	31100 (5135)	43% dec.
2.D_5	35236 (10523)	35% dec.
2.D_10	48024 (13951)	11% dec.
2.D_20	26889 (10667)	50% dec.
2.3_5	14447 (2370)	73% dec.
2.3_10	5684 (1534)	89% dec.
2.3_20	36584 (8308)	32% dec.
2.3.D_7.5	24182 (1547)	55% dec.
2.3.D_15	24564 (3266)	55% dec.
2.3.D_30	34562 (4669)	36% dec.
	**LA4 (SRRC 594)**	
Control	164413 (7179)	N/A
3.D_5	89003 (25905)	46% dec.
3.D_10	105741 (22756)	36% dec.
3.D_20	89681 (33938)	45% dec.
2.D_5	47731 (14367)	71% dec.
2.D_10	62356 (21007)	62% dec.
2.D_20	52341 (8006)	68% dec.
2.3_5	45767 (8293)	72% dec.
2.3_10	39070 (8026)	76% dec.
2.3_20	36550 (8664)	78% dec.
2.3.D_7.5	77114 (12367)	53% dec.
2.3.D_15	100837 (18154)	39% dec.
2.3.D_30	107254 (9236)	35% dec.

[1] VOC combinations (2 = 2,3-dihydrofuran, 3 = 3-octanone, D = decane) and total volumes for each treatment (in µl). Control = toxins detected when no VOC combinations were used. LA1 does not produce this toxin and has been left out of this table.

[2] Average mycotoxin concentrations (ppb) assessed for each VOC treatment. The number in parentheses is the standard deviation from the mean.

[3] Percent (%) change in mycotoxin production from the control value. dec. = decrease; inc. = increase. N/A indicates no value could be assessed.

Table S8. Average AFB_2 concentrations assessed for toxic LA strains and VOC treatments.

Treatment[1]	Concentration (ppb)[2]	Percent Change[3]
	**LA2 (SRRC 573)**	
Control	163 (20)	N/A
3.D_5	43 (22)	74% dec.
3.D_10	69 (18)	58% dec.
3.D_20	97 (80)	40% dec.
2.D_5	119 (15)	27% dec.
2.D_10	131 (40)	20% dec.
2.D_20	142 (94)	13% dec.
2.3_5	40 (11)	75% dec.
2.3_10	112 (40)	31% dec.
2.3_20	85 (25)	48% dec.
2.3.D_7.5	144 (35)	11% dec.
2.3.D_15	160 (82)	1% dec.
2.3.D_30	200 (33)	23% inc.
	**LA3 (SRRC 587)**	
Control	492 (33)	N/A
3.D_5	552 (265)	12% inc.
3.D_10	855 (748)	74% inc.
3.D_20	348 (114)	29% dec.
2.D_5	450 (258)	8% dec.
2.D_10	645 (349)	31% inc.
2.D_20	301 (76)	39% dec.
2.3_5	122 (25)	75% dec.
2.3_10	296 (121)	40% dec.
2.3_20	645 (117)	31% inc.
2.3.D_7.5	182 (21)	63% dec.
2.3.D_15	183 (47)	63% dec.
2.3.D_30	315 (90)	36% dec.
	**LA4 (SRRC 594)**	
Control	3435 (737)	N/A
3.D_5	1947 (624)	44% dec.
3.D_10	2286 (565)	34% dec.
3.D_20	2074 (966)	40% dec.
2.D_5	1200 (380)	65% dec.
2.D_10	1426 (584)	59% dec.
2.D_20	1273 (359)	63% dec.
2.3_5	904 (142)	74% dec.
2.3_10	825 (205)	76% dec.
2.3_20	848 (132)	76% dec.
2.3.D_7.5	1590 (269)	54% dec.
2.3.D_15	2020 (414)	42% dec.
2.3.D_30	2044 (106)	41% dec.

[1] VOC combinations (2 = 2,3-dihydrofuran, 3 = 3-octanone, D = decane) and total volumes for each treatment (in µl). Control = toxins detected when no VOC combinations were used. LA1 does not produce this toxin and has been left out of this table.

[2] Average mycotoxin concentrations (ppb) assessed for each VOC treatment. The number in parentheses is the standard deviation from the mean.

[3] Percent (%) change in mycotoxin production from the control value. dec. = decrease; inc. = increase. N/A indicates no value could be assessed.

Table S9. Average CPA concentrations assessed for toxic LA strains and VOC treatments.

Treatment[1]	Concentration (ppm)[2]	Percent Change[3]
	**LA2 (SRRC 573)**	
Control	1202 (180)	N/A
3.D_5	316 (13)	74% dec.
3.D_10	346 (13)	71% dec.
3.D_20	464 (216)	61% dec.
2.D_5	564 (274)	53% dec.
2.D_10	494 (33)	59% dec.
2.D_20	492 (106)	59% dec.
2.3_5	147 (36)	88% dec.
2.3_10	92 (53)	92% dec.
2.3_20	85 (17)	93% dec.
2.3.D_7.5	828 (324)	31% dec.
2.3.D_15	951 (322)	21% dec.
2.3.D_30	675 (457)	44% dec.
	**LA3 (SRRC 587)**	
Control	16651 (32)	N/A
3.D_5	1439 (303)	91% dec.
3.D_10	1242 (317)	93% dec.
3.D_20	2028 (1279)	88% dec.
2.D_5	5487 (698)	67% dec.
2.D_10	8970 (5286)	46% dec.
2.D_20	6855 (2715)	59% dec.
2.3_5	2040 (266)	88% dec.
2.3_10	2125 (325)	87% dec.
2.3_20	1470 (91)	91% dec.
2.3.D_7.5	3379 (131)	80% dec.
2.3.D_15	3255 (262)	80% dec.
2.3.D_30	3455 (881)	79% dec.
	**LA4 (SRRC 594)**	
Control	5157 (400)	N/A
3.D_5	864 (139)	83% dec.
3.D_10	845 (153)	84% dec.
3.D_20	728 (12)	86% dec.
2.D_5	794 (69)	85% dec.
2.D_10	934 (411)	82% dec.
2.D_20	862 (134)	83% dec.
2.3_5	295 (42)	94% dec.
2.3_10	338 (22)	93% dec.
2.3_20	272 (28)	95% dec.
2.3.D_7.5	1480 (184)	71% dec.
2.3.D_15	4677 (1386)	9% dec.
2.3.D_30	2449 (880)	53% dec.

[1] VOC combinations (2 = 2,3-dihydrofuran, 3 = 3-octanone, D = decane) and total volumes for each treatment (in µl). Control = toxins detected when no VOC combinations were used. LA1 does not produce this toxin and has been left out of this table.

[2] Average mycotoxin concentrations (ppm) assessed for each VOC treatment. The number in parentheses is the standard deviation from the mean.

[3] Percent (%) change in mycotoxin production from the control value. dec. = decrease; inc. = increase. N/A indicates no value could be assessed.

Table S10. Average AFG concentrations assessed for LA4 (SRRC 594) with each VOC treatment.

Treatment[1]	Concentration (ppb)[2]	Percent Change[3]
	**AFG_1**	
Control	109084 (13413)	N/A
3.D_5	21443 (9560)	80% dec.
3.D_10	20896 (8053)	81% dec.
3.D_20	21374 (14456)	80% dec.
2.D_5	29553 (23370)	73% dec.
2.D_10	45493 (15913)	58% dec.
2.D_20	42670 (9930)	61% dec.
2.3_5	25401 (5894)	77% dec.
2.3_10	31540 (4094)	71% dec.
2.3_20	26730 (3302)	76% dec.
2.3.D_7.5	54533 (11268)	50% dec.
2.3.D_15	68752 (13435)	37% dec.
2.3.D_30	74120 (1479)	32% dec.
	**AFG_2**	
Control	2062 (316)	N/A
3.D_5	921 (330)	55% dec.
3.D_10	951 (275)	54% dec.
3.D_20	1001 (590)	51% dec.
2.D_5	835 (358)	60% dec.
2.D_10	1023 (449)	50% dec.
2.D_20	996 (284)	52% dec.
2.3_5	556 (94)	73% dec.
2.3_10	824 (45)	60% dec.
2.3_20	681 (63)	67% dec.
2.3.D_7.5	929 (231)	55% dec.
2.3.D_15	1022 (508)	50% dec.
2.3.D_30	1240 (259)	40% dec.

[1] VOC combinations (2 = 2,3-dihydrofuran, 3 = 3-octanone, D = decane) and total volumes for each treatment (in µl). Control = toxins detected when no VOC combinations were used. LA1-LA3 do not produce this toxin and have been left out of this table.

[2] Average mycotoxin concentrations (ppb) assessed for each VOC treatment. The number in parentheses is the standard deviation from the mean.

[3] Percent (%) change in mycotoxin production from the control value. dec. = decrease; inc. = increase. N/A indicates no value could be assessed.

Table S11. Results of two-way ANOVA and Tukey's test comparing LA1 control IDTs with each VOC combination treatment.

3.D ANOVA	SS (Type III)[a]	DF[b]	MS[c]	F (DFn, DFd)[d]	*P* value
Replicate	276701622	2	138350811	F (2, 6) = 0.3014	0.7504
Treatment	1361236843	3	453745614	F (3, 6) = 0.9884	0.4591
**3.D Tukey's**	**Mean Diff.[e]**	**95.00% CI of diff.[f]**	**Below threshold?**	**Summary[g]**	**Adjusted *P* Value**
Control vs. 3.D_5	17448	-43111 to 78008	No	ns	0.7568
Control vs. 3.D_10	-9304	-69863 to 51255	No	ns	0.9481
Control vs. 3.D_20	13351	-47208 to 73910	No	ns	0.8681
3.D_5 vs. 3.D_10	-26752	-87312 to 33807	No	ns	0.4776
3.D_5 vs. 3.D_20	-4097	-64657 to 56462	No	ns	0.9950
3.D_10 vs. 3.D_20	22655	-37904 to 83214	No	ns	0.5976
**2.D ANOVA**	**SS (Type III)[a]**	**DF[b]**	**MS[c]**	**F (DFn, DFd)[d]**	***P* value**
Replicate	1014637646	2	507318823	F (2, 6) = 2.067	0.2076
Treatment	1240494025	3	413498008	F (3, 6) = 1.685	0.2684
**2.D Tukey's**	**Mean Diff.[e]**	**95.00% CI of diff.[f]**	**Below threshold?**	**Summary[g]**	**Adjusted *P* Value**
Control vs. 2.D_5	9328	-34954 to 53611	No	ns	0.8821
Control vs. 2.D_10	-17018	-61300 to 27265	No	ns	0.5788
Control vs. 2.D_20	-11249	-55532 to 33033	No	ns	0.8157
2.D_5 vs. 2.D_10	-26346	-70628 to 17936	No	ns	0.2659
2.D_5 vs. 2.D_20	-20578	-64860 to 23705	No	ns	0.4401
2.D_10 vs. 2.D_20	5768	-38514 to 50051	No	ns	0.9670
**2.3 ANOVA**	**SS (Type III)[a]**	**DF[b]**	**MS[c]**	**F (DFn, DFd)[d]**	***P* value**
Replicate	60487448	2	30243724	F (2, 6) = 0.1849	0.8358
Treatment	324708270	3	108236090	F (3, 6) = 0.6616	0.6052
**2.3 Tukey's**	**Mean Diff.[e]**	**95.00% CI of diff.[f]**	**Below threshold?**	**Summary[g]**	**Adjusted *P* Value**
Control vs. 2.3_5	-227.0	-36380 to 35926	No	ns	> 0.9999
Control vs. 2.3_10	12399	-23754 to 48552	No	ns	0.6556
Control vs. 2.3_20	1796	-34357 to 37949	No	ns	0.9980
2.3_5 vs. 2.3_10	12626	-23527 to 48779	No	ns	0.6438
2.3_5 vs. 2.3_20	2023	-34130 to 38176	No	ns	0.9971
2.3_10 vs. 2.3_20	-10603	-46756 to 25550	No	ns	0.7475
**2.3.D ANOVA**	**SS (Type III)[a]**	**DF[b]**	**MS[c]**	**F (DFn, DFd)[d]**	***P* value**
Replicate	55611637	2	27805818	F (2, 6) = 0.3937	0.6908
Treatment	2621011424	3	873670475	F (3, 6) = 12.37	0.0056
**2.3.D Tukey's**	**Mean Diff.[e]**	**95.00% CI of diff.[f]**	**Below threshold?**	**Summary[g]**	**Adjusted *P* Value**
Control vs. 2.3.D_7.5	38033	14278 to 61788	Yes	**	0.0059
Control vs. 2.3.D_15	16965	-6790 to 40720	No	ns	0.1625
Control vs. 2.3.D_30	32361	8606 to 56116	Yes	*	0.0129
2.3.D_7.5 vs. 2.3.D_15	-21068	-44823 to 2687	No	ns	0.0791
2.3.D_7.5 vs. 2.3.D_30	-5672	-29427 to 18083	No	ns	0.8404
2.3.D_15 vs. 2.3.D_30	15396	-8359 to 39151	No	ns	0.2139

[a] Partial (non-sequential) Sum of Squares [b] Degrees of Freedom [c] Mean Squares [d] F ratios based on Degrees of Freedom. n = numerator, d = denominator [e] Difference from the control mean. Negative values indicate enhanced growth and positive values indicate reduced growth. [f] Confidence interval (CI) of difference from control [g] Not significant (ns) > 0.05, * $P \leq 0.05$, ** $P \leq 0.01$, *** $P \leq 0.001$, **** $P \leq 0.0001$.

Table S12. Results of two-way ANOVA and Tukey's test comparing LA2 control AFB_1 with each VOC combination treatment.

**3.D ANOVA**	**SS (Type III)**[a]	**DF**[b]	**MS**[c]	**F (DFn, DFd)**[d]	***P* value**
Replicate	42745825	2	21372912	F (2, 6) = 1.184	0.3686
Treatment	585001882	3	195000627	F (3, 6) = 10.80	0.0078
**3.D Tukey's**	**Mean Diff.**[e]	**95.00% CI of diff.**[f]	**Below threshold?**	**Summary**[g]	**Adjusted *P* Value**
Control vs. 3.D_5	17667	5658 to 29676	Yes	**	0.0089
Control vs. 3.D_10	16404	4395 to 28414	Yes	*	0.0127
Control vs. 3.D_20	12342	332.8 to 24351	Yes	*	0.0448
3.D_5 vs. 3.D_10	-1263	-13272 to 10747	No	ns	0.9820
3.D_5 vs. 3.D_20	-5325	-17334 to 6684	No	ns	0.4749
3.D_10 vs. 3.D_20	-4062	-16072 to 7947	No	ns	0.6644
**2.D ANOVA**	**SS (Type III)**[a]	**DF**[b]	**MS**[c]	**F (DFn, DFd)**[d]	***P* value**
Replicate	37611802	2	18805901	F (2, 6) = 1.078	0.3981
Treatment	335499628	3	111833209	F (3, 6) = 6.412	0.0266
**2.D Tukey's**	**Mean Diff.**[e]	**95.00% CI of diff.**[f]	**Below threshold?**	**Summary**[g]	**Adjusted *P* Value**
Control vs. 2.D_5	12374	569.8 to 24178	Yes	*	0.0413
Control vs. 2.D_10	12624	820.1 to 24429	Yes	*	0.0380
Control vs. 2.D_20	11526	-277.9 to 23331	No	ns	0.0549
2.D_5 vs. 2.D_10	250.3	-11554 to 12055	No	ns	0.9998
2.D_5 vs. 2.D_20	-847.7	-12652 to 10957	No	ns	0.9940
2.D_10 vs. 2.D_20	-1098	-12902 to 10706	No	ns	0.9873
**2.3 ANOVA**	**SS (Type III)**[a]	**DF**[b]	**MS**[c]	**F (DFn, DFd)**[d]	***P* value**
Replicate	42756382	2	21378191	F (2, 6) = 11.88	0.0082
Treatment	468904195	3	156301398	F (3, 6) = 86.87	< 0.0001
**2.3 Tukey's**	**Mean Diff.**[e]	**95.00% CI of diff.**[f]	**Below threshold?**	**Summary**[g]	**Adjusted *P* Value**
Control vs. 2.3_5	15942	12151 to 19734	Yes	****	< 0.0001
Control vs. 2.3_10	11483	7692 to 15274	Yes	***	0.0002
Control vs. 2.3_20	14433	10642 to 18225	Yes	****	< 0.0001
2.3_5 vs. 2.3_10	-4459	-8251 to -668.0	Yes	*	0.0253
2.3_5 vs. 2.3_20	-1509	-5300 to 2282	No	ns	0.5539
2.3_10 vs. 2.3_20	2950	-841.0 to 6742	No	ns	0.1243
**2.3.D ANOVA**	**SS (Type III)**[a]	**DF**[b]	**MS**[c]	**F (DFn, DFd)**[d]	***P* value**
Replicate	112813105	2	56406552	F (2, 6) = 2.192	0.1929
Treatment	252564056	3	84188019	F (3, 6) = 3.272	0.1009
**2.3.D Tukey's**	**Mean Diff.**[e]	**95.00% CI of diff.**[f]	**Below threshold?**	**Summary**[g]	**Adjusted *P* Value**
Control vs. 2.3.D_7.5	9257	-5080 to 23595	No	ns	0.2160
Control vs. 2.3.D_15	2747	-11590 to 17084	No	ns	0.9071
Control vs. 2.3.D_30	-3208	-17545 to 11130	No	ns	0.8633
2.3.D_7.5 vs. 2.3.D_15	-6510	-20848 to 7827	No	ns	0.4572
2.3.D_7.5 vs. 2.3.D_30	-12465	-26802 to 1872	No	ns	0.0851
2.3.D_15 vs. 2.3.D_30	-5955	-20292 to 8383	No	ns	0.5231

[a] Partial (non-sequential) Sum of Squares [b] Degrees of Freedom [c] Mean Squares [d] F ratios based on Degrees of Freedom. n = numerator, d = denominator [e] Difference from the control mean. Negative values indicate enhanced growth and positive values indicate reduced growth. [f] Confidence interval (CI) of difference from control [g] Not significant (ns) > 0.05, * $P \leq 0.05$, ** $P \leq 0.01$, *** $P \leq 0.001$, **** $P \leq 0.0001$.

Table S13. Results of two-way ANOVA and Tukey's test comparing LA2 control AFB_2 with each VOC combination treatment.

3.D ANOVA	SS (Type III)[a]	DF[b]	MS[c]	F (DFn, DFd)[d]	*P* value
Replicate	3981	2	1991	F (2, 6) = 1.068	0.4011
Treatment	23936	3	7979	F (3, 6) = 4.280	0.0616
**3.D Tukey's**	**Mean Diff.[e]**	**95.00% CI of diff.[f]**	**Below threshold?**	**Summary[g]**	**Adjusted *P* Value**
Control vs. 3.D_5	120.0	-2.031 to 242.0	No	ns	0.0534
Control vs. 3.D_10	93.67	-28.36 to 215.7	No	ns	0.1300
Control vs. 3.D_20	65.67	-56.36 to 187.7	No	ns	0.3334
3.D_5 vs. 3.D_10	-26.33	-148.4 to 95.70	No	ns	0.8749
3.D_5 vs. 3.D_20	-54.33	-176.4 to 67.70	No	ns	0.4718
3.D_10 vs. 3.D_20	-28.00	-150.0 to 94.03	No	ns	0.8548
**2.D ANOVA**	**SS (Type III)[a]**	**DF[b]**	**MS[c]**	**F (DFn, DFd)[d]**	***P* value**
Replicate	8129	2	4064	F (2, 6) = 1.768	0.2491
Treatment	3082	3	1027	F (3, 6) = 0.4469	0.7285
**2.D Tukey's**	**Mean Diff.[e]**	**95.00% CI of diff.[f]**	**Below threshold?**	**Summary[g]**	**Adjusted *P* Value**
Control vs. 2.D_5	43.33	-92.18 to 178.8	No	ns	0.6988
Control vs. 2.D_10	32.00	-103.5 to 167.5	No	ns	0.8445
Control vs. 2.D_20	20.33	-115.2 to 155.8	No	ns	0.9513
2.D_5 vs. 2.D_10	-11.33	-146.8 to 124.2	No	ns	0.9907
2.D_5 vs. 2.D_20	-23.00	-158.5 to 112.5	No	ns	0.9323
2.D_10 vs. 2.D_20	-11.67	-147.2 to 123.8	No	ns	0.9899
**2.3 ANOVA**	**SS (Type III)[a]**	**DF[b]**	**MS[c]**	**F (DFn, DFd)[d]**	***P* value**
Replicate	3610	2	1805	F (2, 6) = 6.223	0.0344
Treatment	23863	3	7954	F (3, 6) = 27.42	0.0007
**2.3 Tukey's**	**Mean Diff.[e]**	**95.00% CI of diff.[f]**	**Below threshold?**	**Summary[g]**	**Adjusted *P* Value**
Control vs. 2.3_5	123.0	74.86 to 171.1	Yes	***	0.0005
Control vs. 2.3_10	50.33	2.193 to 98.47	Yes	*	0.0417
Control vs. 2.3_20	78.00	29.86 to 126.1	Yes	**	0.0055
2.3_5 vs. 2.3_10	-72.67	-120.8 to -24.53	Yes	**	0.0079
2.3_5 vs. 2.3_20	-45.00	-93.14 to 3.140	No	ns	0.0650
2.3_10 vs. 2.3_20	27.67	-20.47 to 75.81	No	ns	0.2885
**2.3.D ANOVA**	**SS (Type III)[a]**	**DF[b]**	**MS[c]**	**F (DFn, DFd)[d]**	***P* value**
Replicate	9617	2	4808	F (2, 6) = 3.103	0.1188
Treatment	5064	3	1688	F (3, 6) = 1.089	0.4227
**2.3.D Tukey's**	**Mean Diff.[e]**	**95.00% CI of diff.[f]**	**Below threshold?**	**Summary[g]**	**Adjusted *P* Value**
Control vs. 2.3.D_7.5	9257	-5080 to 23595	No	ns	0.2160
Control vs. 2.3.D_15	2747	-11590 to 17084	No	ns	0.9071
Control vs. 2.3.D_30	-3208	-17545 to 11130	No	ns	0.8633
2.3.D_7.5 vs. 2.3.D_15	-6510	-20848 to 7827	No	ns	0.4572
2.3.D_7.5 vs. 2.3.D_30	-12465	-26802 to 1872	No	ns	0.0851
2.3.D_15 vs. 2.3.D_30	-5955	-20292 to 8383	No	ns	0.5231

[a] Partial (non-sequential) Sum of Squares [b] Degrees of Freedom [c] Mean Squares. [d] F ratios based on Degrees of Freedom. n = numerator, d = denominator [e] Difference from the control mean. Negative values indicate enhanced growth and positive values indicate reduced growth. [f] Confidence interval (CI) of difference from control [g] Not significant (ns) > 0.05, * $P \leq 0.05$, ** $P \leq 0.01$, *** $P \leq 0.001$, **** $P \leq 0.0001$

Table S14. Results of two-way ANOVA and Tukey's test comparing LA2 control CPA with each VOC combination treatment.

3.D ANOVA	SS (Type III)[a]	DF[b]	MS[c]	F (DFn, DFd)[d]	*P* value
Replicate	77404	2	38702	F (2, 6) = 2.872	0.1334
Treatment	1575865	3	525288	F (3, 6) = 38.98	0.0002
**3.D Tukey's**	**Mean Diff.[e]**	**95.00% CI of diff.[f]**	**Below threshold?**	**Summary[g]**	**Adjusted *P* Value**
Control vs. 3.D_5	886.7	558.5 to 1215	Yes	***	0.0004
Control vs. 3.D_10	856.3	528.2 to 1184	Yes	***	0.0004
Control vs. 3.D_20	738.0	409.9 to 1066	Yes	***	0.0010
3.D_5 vs. 3.D_10	-30.33	-358.5 to 297.8	No	ns	0.9876
3.D_5 vs. 3.D_20	-148.7	-476.8 to 179.5	No	ns	0.4588
3.D_10 vs. 3.D_20	-118.3	-446.5 to 209.8	No	ns	0.6226
**2.D ANOVA**	**SS (Type III)[a]**	**DF[b]**	**MS[c]**	**F (DFn, DFd)[d]**	***P* value**
Replicate	72892	2	36446	F (2, 6) = 1.311	0.3371
Treatment	1066872	3	355624	F (3, 6) = 12.79	0.0051
**2.D Tukey's**	**Mean Diff.[e]**	**95.00% CI of diff.[f]**	**Below threshold?**	**Summary[g]**	**Adjusted *P* Value**
Control vs. 2.D_5	638.0	166.7 to 1109	Yes	*	0.0133
Control vs. 2.D_10	708.0	236.7 to 1179	Yes	**	0.0080
Control vs. 2.D_20	710.0	238.7 to 1181	Yes	**	0.0079
2.D_5 vs. 2.D_10	70.00	-401.3 to 541.3	No	ns	0.9527
2.D_5 vs. 2.D_20	72.00	-399.3 to 543.3	No	ns	0.9489
2.D_10 vs. 2.D_20	2.000	-469.3 to 473.3	No	ns	> 0.9999
**2.3 ANOVA**	**SS (Type III)[a]**	**DF[b]**	**MS[c]**	**F (DFn, DFd)[d]**	***P* value**
Replicate	35108	2	17554	F (2, 6) = 2.762	0.1411
Treatment	2701427	3	900476	F (3, 6) = 141.7	< 0.0001
**2.3 Tukey's**	**Mean Diff.[e]**	**95.00% CI of diff.[f]**	**Below threshold?**	**Summary[g]**	**Adjusted *P* Value**
Control vs. 2.3_5	1055	830.0 to 1281	Yes	****	< 0.0001
Control vs. 2.3_10	1111	885.3 to 1336	Yes	****	< 0.0001
Control vs. 2.3_20	1117	891.7 to 1342	Yes	****	< 0.0001
2.3_5 vs. 2.3_10	55.33	-170.0 to 280.7	No	ns	0.8295
2.3_5 vs. 2.3_20	61.67	-163.7 to 287.0	No	ns	0.7823
2.3_10 vs. 2.3_20	6.333	-219.0 to 231.7	No	ns	0.9996
**2.3.D ANOVA**	**SS (Type III)[a]**	**DF[b]**	**MS[c]**	**F (DFn, DFd)[d]**	***P* value**
Replicate	276460	2	138230	F (2, 6) = 1.331	0.3324
Treatment	446490	3	148830	F (3, 6) = 1.433	0.3231
**2.3.D Tukey's**	**Mean Diff.[e]**	**95.00% CI of diff.[f]**	**Below threshold?**	**Summary[g]**	**Adjusted *P* Value**
Control vs. 2.3.D_7.5	374.0	-537.0 to 1285	No	ns	0.5315
Control vs. 2.3.D_15	251.0	-660.0 to 1162	No	ns	0.7790
Control vs. 2.3.D_30	527.0	-384.0 to 1438	No	ns	0.2841
2.3.D_7.5 vs. 2.3.D_15	-123.0	-1034 to 788.0	No	ns	0.9636
2.3.D_7.5 vs. 2.3.D_30	153.0	-758.0 to 1064	No	ns	0.9341
2.3.D_15 vs. 2.3.D_30	276.0	-635.0 to 1187	No	ns	0.7298

[a] Partial (non-sequential) Sum of Squares [b] Degrees of Freedom [c] Mean Squares [d] F ratios based on Degrees of Freedom. n = numerator, d = denominator [e] Difference from the control mean. Negative values indicate enhanced growth and positive values indicate reduced growth. [f] Confidence interval (CI) of difference from control [g] Not significant (ns) > 0.05, * $P \leq 0.05$, ** $P \leq 0.01$, *** $P \leq 0.001$, **** $P \leq 0.0001$.

Table S15. Results of two-way ANOVA and Tukey's test comparing LA2 control IDTs with each VOC combination treatment.

**3.D ANOVA**	**SS (Type III)[a]**	**DF[b]**	**MS[c]**	**F (DFn, DFd)[d]**	***P* value**
Replicate	76605	2	38303	F (2, 6) = 1.020	0.4157
Treatment	79319	3	26440	F (3, 6) = 0.7038	0.5836
**3.D Tukey's**	**Mean Diff.[e]**	**95.00% CI of diff.[f]**	**Below threshold?**	**Summary[g]**	**Adjusted *P* Value**
Control vs. 3.D_5	47.67	-500.1 to 595.5	No	ns	0.9896
Control vs. 3.D_10	-142.3	-690.1 to 405.5	No	ns	0.8061
Control vs. 3.D_20	64.33	-483.5 to 612.1	No	ns	0.9753
3.D_5 vs. 3.D_10	-190.0	-737.8 to 357.8	No	ns	0.6483
3.D_5 vs. 3.D_20	16.67	-531.1 to 564.5	No	ns	0.9995
3.D_10 vs. 3.D_20	206.7	-341.1 to 754.5	No	ns	0.5918
**2.D ANOVA**	**SS (Type III)[a]**	**DF[b]**	**MS[c]**	**F (DFn, DFd)[d]**	***P* value**
Replicate	151530	2	75765	F (2, 6) = 2.258	0.1857
Treatment	57095	3	19032	F (3, 6) = 0.5673	0.6566
**2.D Tukey's**	**Mean Diff.[e]**	**95.00% CI of diff.[f]**	**Below threshold?**	**Summary[g]**	**Adjusted *P* Value**
Control vs. 2.D_5	-164.3	-682.0 to 353.4	No	ns	0.7032
Control vs. 2.D_10	-50.33	-568.0 to 467.4	No	ns	0.9856
Control vs. 2.D_20	-152.0	-669.7 to 365.7	No	ns	0.7469
2.D_5 vs. 2.D_10	114.0	-403.7 to 631.7	No	ns	0.8685
2.D_5 vs. 2.D_20	12.33	-505.4 to 530.0	No	ns	0.9998
2.D_10 vs. 2.D_20	-101.7	-619.4 to 416.0	No	ns	0.9011
**2.3 ANOVA**	**SS (Type III)[a]**	**DF[b]**	**MS[c]**	**F (DFn, DFd)[d]**	***P* value**
Replicate	331742	2	165871	F (2, 6) = 0.5763	0.5903
Treatment	414930	3	138310	F (3, 6) = 0.4805	0.7077
**2.3 Tukey's**	**Mean Diff.[e]**	**95.00% CI of diff.[f]**	**Below threshold?**	**Summary[g]**	**Adjusted *P* Value**
Control vs. 2.3_5	-350.3	-1867 to 1166	No	ns	0.8524
Control vs. 2.3_10	-207.0	-1723 to 1309	No	ns	0.9625
Control vs. 2.3_20	-504.7	-2021 to 1012	No	ns	0.6746
2.3_5 vs. 2.3_10	143.3	-1373 to 1660	No	ns	0.9867
2.3_5 vs. 2.3_20	-154.3	-1671 to 1362	No	ns	0.9836
2.3_10 vs. 2.3_20	-297.7	-1814 to 1219	No	ns	0.9012
**2.3.D ANOVA**	**SS (Type III)[a]**	**DF[b]**	**MS[c]**	**F (DFn, DFd)[d]**	***P* value**
Replicate	75446	2	37723	F (2, 6) = 13.12	0.0065
Treatment	20657	3	6886	F (3, 6) = 2.394	0.1670
**2.3.D Tukey's**	**Mean Diff.[e]**	**95.00% CI of diff.[f]**	**Below threshold?**	**Summary[g]**	**Adjusted *P* Value**
Control vs. 2.3.D_7.5	38.00	-113.6 to 189.6	No	ns	0.8212
Control vs. 2.3.D_15	36.67	-114.9 to 188.2	No	ns	0.8354
Control vs. 2.3.D_30	114.0	-37.58 to 265.6	No	ns	0.1387
2.3.D_7.5 vs. 2.3.D_15	-1.333	-152.9 to 150.2	No	ns	> 0.9999
2.3.D_7.5 vs. 2.3.D_30	76.00	-75.58 to 227.6	No	ns	0.3841
2.3.D_15 vs. 2.3.D_30	77.33	-74.25 to 228.9	No	ns	0.3715

[a] Partial (non-sequential) Sum of Squares [b] Degrees of Freedom [c] Mean Squares [d] F ratios based on Degrees of Freedom. n = numerator, d = denominator [e] Difference from the control mean. Negative values indicate enhanced growth and positive values indicate reduced growth. [f] Confidence interval (CI) of difference from control [g] Not significant (ns) > 0.05, * $P \leq 0.05$, ** $P \leq 0.01$, *** $P \leq 0.001$, **** $P \leq 0.0001$.

Table S16. Results of two-way ANOVA and Tukey's test comparing LA3 control AFB_1 with each VOC combination treatment.

3.D ANOVA	SS (Type III)[a]	DF[b]	MS[c]	F (DFn, DFd)[d]	*P* value
Replicate	475549536	2	237774768	F (2, 6) = 1.668	0.2654
Treatment	1038355569	3	346118523	F (3, 6) = 2.429	0.1635
**3.D Tukey's**	**Mean Diff.[e]**	**95.00% CI of diff.[f]**	**Below threshold?**	**Summary[g]**	**Adjusted *P* Value**
Control vs. 3.D_5	12376	-21367 to 46119	No	ns	0.6112
Control vs. 3.D_10	1365	-32378 to 35108	No	ns	0.9989
Control vs. 3.D_20	22984	-10759 to 56727	No	ns	0.1865
3.D_5 vs. 3.D_10	-11011	-44754 to 22732	No	ns	0.6867
3.D_5 vs. 3.D_20	10609	-23134 to 44352	No	ns	0.7088
3.D_10 vs. 3.D_20	21619	-12124 to 55362	No	ns	0.2205
**2.D ANOVA**	**SS (Type III)[a]**	**DF[b]**	**MS[c]**	**F (DFn, DFd)[d]**	***P* value**
Replicate	486247842	2	243123921	F (2, 6) = 3.557	0.0958
Treatment	1358614690	3	452871563	F (3, 6) = 6.626	0.0248
**2.D Tukey's**	**Mean Diff.[e]**	**95.00% CI of diff.[f]**	**Below threshold?**	**Summary[g]**	**Adjusted *P* Value**
Control vs. 2.D_5	18848	-4518 to 42215	No	ns	0.1104
Control vs. 2.D_10	6061	-17306 to 29427	No	ns	0.8068
Control vs. 2.D_20	27196	3829 to 50562	Yes	*	0.0265
2.D_5 vs. 2.D_10	-12788	-36154 to 10579	No	ns	0.3217
2.D_5 vs. 2.D_20	8347	-15019 to 31714	No	ns	0.6289
2.D_10 vs. 2.D_20	21135	-2232 to 44502	No	ns	0.0736
**2.3 ANOVA**	**SS (Type III)[a]**	**DF[b]**	**MS[c]**	**F (DFn, DFd)[d]**	***P* value**
Replicate	42346407	2	21173204	F (2, 6) = 0.7489	0.5125
Treatment	4306249543	3	1435416514	F (3, 6) = 50.77	0.0001
**2.3 Tukey's**	**Mean Diff.[e]**	**95.00% CI of diff.[f]**	**Below threshold?**	**Summary[g]**	**Adjusted *P* Value**
Control vs. 2.3_5	39638	24609 to 54667	Yes	***	0.0004
Control vs. 2.3_10	48400	33371 to 63429	Yes	***	0.0001
Control vs. 2.3_20	17501	2472 to 32530	Yes	*	0.0264
2.3_5 vs. 2.3_10	8762	-6267 to 23791	No	ns	0.2790
2.3_5 vs. 2.3_20	-22137	-37166 to -7108	Yes	**	0.0089
2.3_10 vs. 2.3_20	-30900	-45929 to -15871	Yes	**	0.0016
**2.3.D ANOVA**	**SS (Type III)[a]**	**DF[b]**	**MS[c]**	**F (DFn, DFd)[d]**	***P* value**
Replicate	45494100	2	22747050	F (2, 6) = 1.660	0.2668
Treatment	1765927821	3	588642607	F (3, 6) = 42.96	0.0002
**2.3.D Tukey's**	**Mean Diff.[e]**	**95.00% CI of diff.[f]**	**Below threshold?**	**Summary[g]**	**Adjusted *P* Value**
Control vs. 2.3.D_7.5	29902	19439 to 40365	Yes	***	0.0003
Control vs. 2.3.D_15	29521	19058 to 39983	Yes	***	0.0003
Control vs. 2.3.D_30	19522	9060 to 29985	Yes	**	0.0027
2.3.D_7.5 vs. 2.3.D_15	-381.3	-10844 to 10081	No	ns	0.9992
2.3.D_7.5 vs. 2.3.D_30	-10380	-20842 to 83.11	No	ns	0.0516
2.3.D_15 vs. 2.3.D_30	-9998	-20461 to 464.4	No	ns	0.0598

[a] Partial (non-sequential) Sum of Squares [b] Degrees of Freedom [c] Mean Squares [d] F ratios based on Degrees of Freedom. n = numerator, d = denominator [e] Difference from the control mean. Negative values indicate enhanced growth and positive values indicate reduced growth. [f] Confidence interval (CI) of difference from control [g] Not significant (ns) > 0.05, * $P \leq 0.05$, ** $P \leq 0.01$, *** $P \leq 0.001$, **** $P \leq 0.0001$.

Table S17. Results of two-way ANOVA and Tukey's test comparing LA3 control AFB_2 with each VOC combination treatment.

3.D ANOVA	SS (Type III)[a]	DF[b]	MS[c]	F (DFn, DFd)[d]	*P* value
Replicate	581826	2	290913	F (2, 6) = 2.473	0.1647
Treatment	409061	3	136354	F (3, 6) = 1.159	0.3997
**3.D Tukey's**	**Mean Diff.[e]**	**95.00% CI of diff.[f]**	**Below threshold?**	**Summary[g]**	**Adjusted *P* Value**
Control vs. 3.D_5	-60.33	-1030 to 909.2	No	ns	0.9961
Control vs. 3.D_10	-363.0	-1332 to 606.5	No	ns	0.5970
Control vs. 3.D_20	143.3	-826.2 to 1113	No	ns	0.9533
3.D_5 vs. 3.D_10	-302.7	-1272 to 666.8	No	ns	0.7129
3.D_5 vs. 3.D_20	203.7	-765.8 to 1173	No	ns	0.8829
3.D_10 vs. 3.D_20	506.3	-463.2 to 1476	No	ns	0.3546
**2.D ANOVA**	**SS (Type III)[a]**	**DF[b]**	**MS[c]**	**F (DFn, DFd)[d]**	***P* value**
Replicate	204261	2	102131	F (2, 6) = 3.282	0.1089
Treatment	179733	3	59911	F (3, 6) = 1.925	0.2267
**2.D Tukey's**	**Mean Diff.[e]**	**95.00% CI of diff.[f]**	**Below threshold?**	**Summary[g]**	**Adjusted *P* Value**
Control vs. 2.D_5	41.33	-457.3 to 539.9	No	ns	0.9909
Control vs. 2.D_10	-153.0	-651.6 to 345.6	No	ns	0.7227
Control vs. 2.D_20	190.7	-307.9 to 689.3	No	ns	0.5823
2.D_5 vs. 2.D_10	-194.3	-692.9 to 304.3	No	ns	0.5689
2.D_5 vs. 2.D_20	149.3	-349.3 to 647.9	No	ns	0.7362
2.D_10 vs. 2.D_20	343.7	-154.9 to 842.3	No	ns	0.1803
**2.3 ANOVA**	**SS (Type III)[a]**	**DF[b]**	**MS[c]**	**F (DFn, DFd)[d]**	***P* value**
Replicate	26152	2	13076	F (2, 6) = 2.305	0.1809
Treatment	469623	3	156541	F (3, 6) = 27.59	0.0007
**2.3 Tukey's**	**Mean Diff.[e]**	**95.00% CI of diff.[f]**	**Below threshold?**	**Summary[g]**	**Adjusted *P* Value**
Control vs. 2.3_5	370.3	157.4 to 583.2	Yes	**	0.0038
Control vs. 2.3_10	195.7	-17.23 to 408.6	No	ns	0.0694
Control vs. 2.3_20	-153.7	-366.6 to 59.23	No	ns	0.1574
2.3_5 vs. 2.3_10	-174.7	-387.6 to 38.23	No	ns	0.1042
2.3_5 vs. 2.3_20	-524.0	-736.9 to -311.1	Yes	***	0.0006
2.3_10 vs. 2.3_20	-349.3	-562.2 to -136.4	Yes	**	0.0052
**2.3.D ANOVA**	**SS (Type III)[a]**	**DF[b]**	**MS[c]**	**F (DFn, DFd)[d]**	***P* value**
Replicate	4578	2	2289	F (2, 6) = 0.7226	0.5234
Treatment	193296	3	64432	F (3, 6) = 20.34	0.0015
**2.3.D Tukey's**	**Mean Diff.[e]**	**95.00% CI of diff.[f]**	**Below threshold?**	**Summary[g]**	**Adjusted *P* Value**
Control vs. 2.3.D_7.5	309.7	150.6 to 468.7	Yes	**	0.0021
Control vs. 2.3.D_15	309.0	149.9 to 468.1	Yes	**	0.0022
Control vs. 2.3.D_30	177.0	17.92 to 336.1	Yes	*	0.0322
2.3.D_7.5 vs. 2.3.D_15	-0.6667	-159.7 to 158.4	No	ns	> 0.9999
2.3.D_7.5 vs. 2.3.D_30	-132.7	-291.7 to 26.41	No	ns	0.0985
2.3.D_15 vs. 2.3.D_30	-132.0	-291.1 to 27.08	No	ns	0.1002

[a] Partial (non-sequential) Sum of Squares [b] Degrees of Freedom [c] Mean Squares [d] F ratios based on Degrees of Freedom. n = numerator, d = denominator [e] Difference from the control mean. Negative values indicate enhanced growth and positive values indicate reduced growth. [f] Confidence interval (CI) of difference from control [g] Not significant (ns) > 0.05, * $P \leq 0.05$, ** $P \leq 0.01$, *** $P \leq 0.001$, **** $P \leq 0.0001$.

Table S18. Results of two-way ANOVA and Tukey's test comparing LA3 control CPA with each VOC combination treatment.

**3.D ANOVA**	**SS (Type III)[a]**	**DF[b]**	**MS[c]**	**F (DFn, DFd)[d]**	***P* value**
Replicate	909573	2	454786	F (2, 6) = 0.9924	0.4243
Treatment	512795459	3	170931820	F (3, 6) = 373.0	< 0.0001
**3.D Tukey's**	**Mean Diff.[e]**	**95.00% CI of diff.[f]**	**Below threshold?**	**Summary[g]**	**Adjusted *P* Value**
Control vs. 3.D_5	15213	13299 to 17126	Yes	****	< 0.0001
Control vs. 3.D_10	15409	13496 to 17323	Yes	****	< 0.0001
Control vs. 3.D_20	14624	12710 to 16537	Yes	****	< 0.0001
3.D_5 vs. 3.D_10	196.7	-1717 to 2110	No	ns	0.9831
3.D_5 vs. 3.D_20	-589.0	-2502 to 1324	No	ns	0.7209
3.D_10 vs. 3.D_20	-785.7	-2699 to 1128	No	ns	0.5314
**2.D ANOVA**	**SS (Type III)[a]**	**DF[b]**	**MS[c]**	**F (DFn, DFd)[d]**	***P* value**
Replicate	37566115	2	18783058	F (2, 6) = 3.311	0.1074
Treatment	223581300	3	74527100	F (3, 6) = 13.14	0.0048
**2.D Tukey's**	**Mean Diff.[e]**	**95.00% CI of diff.[f]**	**Below threshold?**	**Summary[g]**	**Adjusted *P* Value**
Control vs. 2.D_5	11165	4433 to 17897	Yes	**	0.0049
Control vs. 2.D_10	7682	949.7 to 14414	Yes	*	0.0288
Control vs. 2.D_20	9797	3065 to 16529	Yes	**	0.0094
2.D_5 vs. 2.D_10	-3483	-10215 to 3249	No	ns	0.3613
2.D_5 vs. 2.D_20	-1368	-8100 to 5364	No	ns	0.8922
2.D_10 vs. 2.D_20	2115	-4617 to 8847	No	ns	0.7092
**2.3 ANOVA**	**SS (Type III)[a]**	**DF[b]**	**MS[c]**	**F (DFn, DFd)[d]**	***P* value**
Replicate	238222	2	119111	F (2, 6) = 5.370	0.0460
Treatment	491798838	3	163932946	F (3, 6) = 7391	< 0.0001
**2.3 Tukey's**	**Mean Diff.[e]**	**95.00% CI of diff.[f]**	**Below threshold?**	**Summary[g]**	**Adjusted *P* Value**
Control vs. 2.3_5	14611	14190 to 15032	Yes	****	< 0.0001
Control vs. 2.3_10	14526	14105 to 14947	Yes	****	< 0.0001
Control vs. 2.3_20	15182	14761 to 15603	Yes	****	< 0.0001
2.3_5 vs. 2.3_10	-85.00	-506.0 to 336.0	No	ns	0.8939
2.3_5 vs. 2.3_20	570.7	149.7 to 991.6	Yes	*	0.0132
2.3_10 vs. 2.3_20	655.7	234.7 to 1077	Yes	**	0.0067
**2.3.D ANOVA**	**SS (Type III)[a]**	**DF[b]**	**MS[c]**	**F (DFn, DFd)[d]**	***P* value**
Replicate	751745	2	375872	F (2, 6) = 2.317	0.1796
Treatment	397372169	3	132457390	F (3, 6) = 816.6	< 0.0001
**2.3.D Tukey's**	**Mean Diff.[e]**	**95.00% CI of diff.[f]**	**Below threshold?**	**Summary[g]**	**Adjusted *P* Value**
Control vs. 2.3.D_7.5	13272	12134 to 14411	Yes	****	< 0.0001
Control vs. 2.3.D_15	13396	12258 to 14535	Yes	****	< 0.0001
Control vs. 2.3.D_30	13197	12058 to 14335	Yes	****	< 0.0001
2.3.D_7.5 vs. 2.3.D_15	124.0	-1014 to 1262	No	ns	0.9801
2.3.D_7.5 vs. 2.3.D_30	-75.67	-1214 to 1063	No	ns	0.9952
2.3.D_15 vs. 2.3.D_30	-199.7	-1338 to 938.7	No	ns	0.9261

[a] Partial (non-sequential) Sum of Squares [b] Degrees of Freedom [c] Mean Squares [d] F ratios based on Degrees of Freedom. n = numerator, d = denominator [e] Difference from the control mean. Negative values indicate enhanced growth and positive values indicate reduced growth. [f] Confidence interval (CI) of difference from control [g] Not significant (ns) > 0.05, * $P \leq 0.05$, ** $P \leq 0.01$, *** $P \leq 0.001$, **** $P \leq 0.0001$.

Table S19. Results of two-way ANOVA and Tukey's test comparing LA3 control IDTs with each VOC combination treatment.

**3.D ANOVA**	**SS (Type III)[a]**	**DF[b]**	**MS[c]**	**F (DFn, DFd)[d]**	***P* value**
Replicate	1711754773	2	855877387	F (2, 6) = 4.229	0.0715
Treatment	3665077303	3	1221692434	F (3, 6) = 6.037	0.0304
**3.D Tukey's**	**Mean Diff.[e]**	**95.00% CI of diff.[f]**	**Below threshold?**	**Summary[g]**	**Adjusted *P* Value**
Control vs. 3.D_5	-48769	-88978 to -8560	Yes	*	0.0220
Control vs. 3.D_10	-31359	-71568 to 8851	No	ns	0.1234
Control vs. 3.D_20	-26429	-66639 to 13780	No	ns	0.2059
3.D_5 vs. 3.D_10	17410	-22799 to 57620	No	ns	0.4925
3.D_5 vs. 3.D_20	22340	-17870 to 62549	No	ns	0.3113
3.D_10 vs. 3.D_20	4929	-35280 to 45139	No	ns	0.9722
**2.D ANOVA**	**SS (Type III)[a]**	**DF[b]**	**MS[c]**	**F (DFn, DFd)[d]**	***P* value**
Replicate	2312380580	2	1156190290	F (2, 6) = 1.351	0.3278
Treatment	5359199255	3	1786399752	F (3, 6) = 2.087	0.2034
**2.D Tukey's**	**Mean Diff.[e]**	**95.00% CI of diff.[f]**	**Below threshold?**	**Summary[g]**	**Adjusted *P* Value**
Control vs. 2.D_5	-46742	-129433 to 35949	No	ns	0.2996
Control vs. 2.D_10	-53645	-136336 to 29046	No	ns	0.2133
Control vs. 2.D_20	-22943	-105634 to 59748	No	ns	0.7757
2.D_5 vs. 2.D_10	-6902	-89593 to 75789	No	ns	0.9907
2.D_5 vs. 2.D_20	23799	-58892 to 106490	No	ns	0.7573
2.D_10 vs. 2.D_20	30701	-51990 to 113392	No	ns	0.6028
**2.3 ANOVA**	**SS (Type III)[a]**	**DF[b]**	**MS[c]**	**F (DFn, DFd)[d]**	***P* value**
Replicate	2405430118	2	1202715059	F (2, 6) = 0.9115	0.4512
Treatment	7365004914	3	2455001638	F (3, 6) = 1.860	0.2371
**2.3 Tukey's**	**Mean Diff.[e]**	**95.00% CI of diff.[f]**	**Below threshold?**	**Summary[g]**	**Adjusted *P* Value**
Control vs. 2.3_5	-68381	-171054 to 34293	No	ns	0.1986
Control vs. 2.3_10	-21860	-124534 to 80814	No	ns	0.8789
Control vs. 2.3_20	-25507	-128181 to 77167	No	ns	0.8249
2.3_5 vs. 2.3_10	46521	-56153 to 149194	No	ns	0.4588
2.3_5 vs. 2.3_20	42874	-59800 to 145547	No	ns	0.5191
2.3_10 vs. 2.3_20	-3647	-106321 to 99027	No	ns	0.9993
**2.3.D ANOVA**	**SS (Type III)[a]**	**DF[b]**	**MS[c]**	**F (DFn, DFd)[d]**	***P* value**
Replicate	796390435	2	398195218	F (2, 6) = 1.427	0.3112
Treatment	1487852804	3	495950935	F (3, 6) = 1.778	0.2512
**2.3.D Tukey's**	**Mean Diff.[e]**	**95.00% CI of diff.[f]**	**Below threshold?**	**Summary[g]**	**Adjusted *P* Value**
Control vs. 2.3.D_7.5	-28367	-75579 to 18845	No	ns	0.2596
Control vs. 2.3.D_15	-22808	-70020 to 24404	No	ns	0.4113
Control vs. 2.3.D_30	-24720	-71932 to 22492	No	ns	0.3528
2.3.D_7.5 vs. 2.3.D_15	5559	-41653 to 52771	No	ns	0.9752
2.3.D_7.5 vs. 2.3.D_30	3647	-43565 to 50859	No	ns	0.9926
2.3.D_15 vs. 2.3.D_30	-1912	-49124 to 45300	No	ns	0.9989

[a] Partial (non-sequential) Sum of Squares [b] Degrees of Freedom [c] Mean Squares [d] F ratios based on Degrees of Freedom. n = numerator, d = denominator [e] Difference from the control mean. Negative values indicate enhanced growth and positive values indicate reduced growth. [f] Confidence interval (CI) of difference from control [g] Not significant (ns) > 0.05, * $P \leq 0.05$, ** $P \leq 0.01$, *** $P \leq 0.001$, **** $P \leq 0.0001$.

Table S20. Results of two-way ANOVA and Tukey's test comparing LA4 control AFB_1 with each VOC combination treatment.

3.D ANOVA	SS (Type III)[a]	DF[b]	MS[c]	F (DFn, DFd)[d]	*P* value
Replicate	935324193	2	467662097	F (2, 6) = 0.7290	0.5207
Treatment	11439353997	3	3813117999	F (3, 6) = 5.944	0.0314
**3.D Tukey's**	**Mean Diff.[e]**	**95.00% CI of diff.[f]**	**Below threshold?**	**Summary[g]**	**Adjusted *P* Value**
Control vs. 3.D_5	75410	3820 to 147000	Yes	*	0.0405
Control vs. 3.D_10	58672	-12918 to 130262	No	ns	0.1046
Control vs. 3.D_20	74732	3142 to 146322	Yes	*	0.0420
3.D_5 vs. 3.D_10	-16739	-88329 to 54851	No	ns	0.8481
3.D_5 vs. 3.D_20	-678.7	-72269 to 70911	No	ns	> 0.9999
3.D_10 vs. 3.D_20	16060	-55530 to 87650	No	ns	0.8625
**2.D ANOVA**	**SS (Type III)[a]**	**DF[b]**	**MS[c]**	**F (DFn, DFd)[d]**	***P* value**
Replicate	234845805	2	117422903	F (2, 6) = 0.5454	0.6059
Treatment	27694324934	3	9231441645	F (3, 6) = 42.88	0.0002
**2.D Tukey's**	**Mean Diff.[e]**	**95.00% CI of diff.[f]**	**Below threshold?**	**Summary[g]**	**Adjusted *P* Value**
Control vs. 2.D_5	116682	75208 to 158156	Yes	***	0.0003
Control vs. 2.D_10	102057	60583 to 143530	Yes	***	0.0006
Control vs. 2.D_20	112072	70598 to 153545	Yes	***	0.0004
2.D_5 vs. 2.D_10	-14625	-56099 to 26848	No	ns	0.6375
2.D_5 vs. 2.D_20	-4610	-46084 to 36863	No	ns	0.9789
2.D_10 vs. 2.D_20	10015	-31459 to 51489	No	ns	0.8361
**2.3 ANOVA**	**SS (Type III)[a]**	**DF[b]**	**MS[c]**	**F (DFn, DFd)[d]**	***P* value**
Replicate	210896352	2	105448176	F (2, 6) = 2.050	0.2097
Treatment	34704751940	3	11568250647	F (3, 6) = 224.9	< 0.0001
**2.3 Tukey's**	**Mean Diff.[e]**	**95.00% CI of diff.[f]**	**Below threshold?**	**Summary[g]**	**Adjusted *P* Value**
Control vs. 2.3_5	118646	98373 to 138919	Yes	****	< 0.0001
Control vs. 2.3_10	125343	105070 to 145616	Yes	****	< 0.0001
Control vs. 2.3_20	127863	107590 to 148136	Yes	****	< 0.0001
2.3_5 vs. 2.3_10	6697	-13576 to 26970	No	ns	0.6792
2.3_5 vs. 2.3_20	9217	-11056 to 29490	No	ns	0.4563
2.3_10 vs. 2.3_20	2520	-17753 to 22793	No	ns	0.9711
**2.3.D ANOVA**	**SS (Type III)[a]**	**DF[b]**	**MS[c]**	**F (DFn, DFd)[d]**	***P* value**
Replicate	14481661	2	7240830	F (2, 6) = 0.03549	0.9653
Treatment	12331820791	3	4110606930	F (3, 6) = 20.15	0.0016
**2.3.D Tukey's**	**Mean Diff.[e]**	**95.00% CI of diff.[f]**	**Below threshold?**	**Summary[g]**	**Adjusted *P* Value**
Control vs. 2.3.D_7.5	87299	46925 to 127673	Yes	**	0.0012
Control vs. 2.3.D_15	63576	23202 to 103950	Yes	**	0.0064
Control vs. 2.3.D_30	57159	16785 to 97533	Yes	*	0.0107
2.3.D_7.5 vs. 2.3.D_15	-23723	-64097 to 16651	No	ns	0.2739
2.3.D_7.5 vs. 2.3.D_30	-30140	-70514 to 10234	No	ns	0.1419
2.3.D_15 vs. 2.3.D_30	-6417	-46791 to 33957	No	ns	0.9432

[a] Partial (non-sequential) Sum of Squares [b] Degrees of Freedom [c] Mean Squares [d] F ratios based on Degrees of Freedom. n = numerator, d = denominator [e] Difference from the control mean. Negative values indicate enhanced growth and positive values indicate reduced growth. [f] Confidence interval (CI) of difference from control [g] Not significant (ns) > 0.05, * $P \le 0.05$, ** $P \le 0.01$, *** $P \le 0.001$, **** $P \le 0.0001$.

Table S21. Results of two-way ANOVA and Tukey's test comparing LA4 control AFB_2 with each VOC combination treatment.

**3.D ANOVA**	**SS (Type III)[a]**	**DF[b]**	**MS[c]**	**F (DFn, DFd)[d]**	***P* value**
Replicate	1863006	2	931503	F (2, 6) = 2.072	0.2070
Treatment	4392920	3	1464307	F (3, 6) = 3.256	0.1017
**3.D Tukey's**	**Mean Diff.[e]**	**95.00% CI of diff.[f]**	**Below threshold?**	**Summary[g]**	**Adjusted *P* Value**
Control vs. 3.D_5	1524	-371.4 to 3419	No	ns	0.1116
Control vs. 3.D_10	1186	-709.7 to 3081	No	ns	0.2347
Control vs. 3.D_20	1398	-497.7 to 3293	No	ns	0.1474
3.D_5 vs. 3.D_10	-338.3	-2234 to 1557	No	ns	0.9227
3.D_5 vs. 3.D_20	-126.3	-2022 to 1769	No	ns	0.9952
3.D_10 vs. 3.D_20	212.0	-1683 to 2107	No	ns	0.9785
**2.D ANOVA**	**SS (Type III)[a]**	**DF[b]**	**MS[c]**	**F (DFn, DFd)[d]**	***P* value**
Replicate	22261	2	11131	F (2, 6) = 0.02687	0.9736
Treatment	10689835	3	3563278	F (3, 6) = 8.601	0.0136
**2.D Tukey's**	**Mean Diff.[e]**	**95.00% CI of diff.[f]**	**Below threshold?**	**Summary[g]**	**Adjusted *P* Value**
Control vs. 2.D_5	2271	451.7 to 4090	Yes	*	0.0194
Control vs. 2.D_10	2045	226.0 to 3865	Yes	*	0.0308
Control vs. 2.D_20	2198	379.0 to 4018	Yes	*	0.0224
2.D_5 vs. 2.D_10	-225.7	-2045 to 1594	No	ns	0.9712
2.D_5 vs. 2.D_20	-72.67	-1892 to 1747	No	ns	0.9989
2.D_10 vs. 2.D_20	153.0	-1666 to 1972	No	ns	0.9905
**2.3 ANOVA**	**SS (Type III)[a]**	**DF[b]**	**MS[c]**	**F (DFn, DFd)[d]**	***P* value**
Replicate	106840	2	53420	F (2, 6) = 0.2407	0.7933
Treatment	15368467	3	5122822	F (3, 6) = 23.08	0.0011
**2.3 Tukey's**	**Mean Diff.[e]**	**95.00% CI of diff.[f]**	**Below threshold?**	**Summary[g]**	**Adjusted *P* Value**
Control vs. 2.3_5	2568	1236 to 3899	Yes	**	0.0022
Control vs. 2.3_10	2647	1315 to 3978	Yes	**	0.0019
Control vs. 2.3_20	2624	1292 to 3955	Yes	**	0.0020
2.3_5 vs. 2.3_10	79.00	-1253 to 1411	No	ns	0.9966
2.3_5 vs. 2.3_20	56.00	-1276 to 1388	No	ns	0.9988
2.3_10 vs. 2.3_20	-23.00	-1355 to 1309	No	ns	> 0.9999
**2.3.D ANOVA**	**SS (Type III)[a]**	**DF[b]**	**MS[c]**	**F (DFn, DFd)[d]**	***P* value**
Replicate	405227	2	202613	F (2, 6) = 0.8784	0.4628
Treatment	6055608	3	2018536	F (3, 6) = 8.751	0.0131
**2.3.D Tukey's**	**Mean Diff.[e]**	**95.00% CI of diff.[f]**	**Below threshold?**	**Summary[g]**	**Adjusted *P* Value**
Control vs. 2.3.D_7.5	1881	523.5 to 3238	Yes	*	0.0119
Control vs. 2.3.D_15	1452	94.21 to 2809	Yes	*	0.0380
Control vs. 2.3.D_30	1428	70.21 to 2785	Yes	*	0.0407
2.3.D_7.5 vs. 2.3.D_15	-429.3	-1787 to 928.1	No	ns	0.7053
2.3.D_7.5 vs. 2.3.D_30	-453.3	-1811 to 904.1	No	ns	0.6724
2.3.D_15 vs. 2.3.D_30	-24.00	-1381 to 1333	No	ns	> 0.9999

[a] Partial (non-sequential) Sum of Squares [b] Degrees of Freedom [c] Mean Squares [d] F ratios based on Degrees of Freedom. n = numerator, d = denominator [e] Difference from the control mean. Negative values indicate enhanced growth and positive values indicate reduced growth. [f] Confidence interval (CI) of difference from control [g] Not significant (ns) > 0.05, * $P \leq 0.05$, ** $P \leq 0.01$, *** $P \leq 0.001$, **** $P \leq 0.0001$.

Table S22. Results of two-way ANOVA and Tukey's test comparing LA4 control AFG_1 with each VOC combination treatment.

3.D ANOVA	SS (Type III)[a]	DF[b]	MS[c]	F (DFn, DFd)[d]	*P* value
Replicate	383900665	2	191950332	F (2, 6) = 1.630	0.2720
Treatment	17363865354	3	5787955118	F (3, 6) = 49.16	0.0001
**3.D Tukey's**	**Mean Diff.[e]**	**95.00% CI of diff.[f]**	**Below threshold?**	**Summary[g]**	**Adjusted *P* Value**
Control vs. 3.D_5	87641	56973 to 118309	Yes	***	0.0003
Control vs. 3.D_10	88188	57520 to 118856	Yes	***	0.0002
Control vs. 3.D_20	87710	57042 to 118378	Yes	***	0.0003
3.D_5 vs. 3.D_10	547.0	-30121 to 31215	No	ns	> 0.9999
3.D_5 vs. 3.D_20	69.00	-30599 to 30737	No	ns	> 0.9999
3.D_10 vs. 3.D_20	-478.0	-31146 to 30190	No	ns	> 0.9999
**2.D ANOVA**	**SS (Type III)[a]**	**DF[b]**	**MS[c]**	**F (DFn, DFd)[d]**	***P* value**
Replicate	92802172	2	46401086	F (2, 6) = 0.1350	0.8763
Treatment	11410487271	3	3803495757	F (3, 6) = 11.06	0.0074
**2.D Tukey's**	**Mean Diff.[e]**	**95.00% CI of diff.[f]**	**Below threshold?**	**Summary[g]**	**Adjusted *P* Value**
Control vs. 2.D_5	79531	27121 to 131941	Yes	**	0.0077
Control vs. 2.D_10	63592	11181 to 116002	Yes	*	0.0220
Control vs. 2.D_20	66414	14004 to 118824	Yes	*	0.0181
2.D_5 vs. 2.D_10	-15939	-68350 to 36471	No	ns	0.7277
2.D_5 vs. 2.D_20	-13117	-65527 to 39293	No	ns	0.8219
2.D_10 vs. 2.D_20	2822	-49588 to 55233	No	ns	0.9974
**2.3 ANOVA**	**SS (Type III)[a]**	**DF[b]**	**MS[c]**	**F (DFn, DFd)[d]**	***P* value**
Replicate	39512712	2	19756356	F (2, 6) = 0.2663	0.7748
Treatment	14895637343	3	4965212448	F (3, 6) = 66.93	< 0.0001
**2.3 Tukey's**	**Mean Diff.[e]**	**95.00% CI of diff.[f]**	**Below threshold?**	**Summary[g]**	**Adjusted *P* Value**
Control vs. 2.3_5	83683	59338 to 108028	Yes	****	< 0.0001
Control vs. 2.3_10	77544	53199 to 101889	Yes	***	0.0001
Control vs. 2.3_20	82354	58009 to 106699	Yes	****	< 0.0001
2.3_5 vs. 2.3_10	-6139	-30484 to 18206	No	ns	0.8188
2.3_5 vs. 2.3_20	-1329	-25674 to 23016	No	ns	0.9973
2.3_10 vs. 2.3_20	4810	-19535 to 29155	No	ns	0.8996
**2.3.D ANOVA**	**SS (Type III)[a]**	**DF[b]**	**MS[c]**	**F (DFn, DFd)[d]**	***P* value**
Replicate	149500862	2	74750431	F (2, 6) = 0.5406	0.6083
Treatment	4829722547	3	1609907516	F (3, 6) = 11.64	0.0065
**2.3.D Tukey's**	**Mean Diff.[e]**	**95.00% CI of diff.[f]**	**Below threshold?**	**Summary[g]**	**Adjusted *P* Value**
Control vs. 2.3.D_7.5	54551	21315 to 87787	Yes	**	0.0052
Control vs. 2.3.D_15	40332	7096 to 73569	Yes	*	0.0220
Control vs. 2.3.D_30	34964	1728 to 68200	Yes	*	0.0407
2.3.D_7.5 vs. 2.3.D_15	-14219	-47455 to 19018	No	ns	0.5014
2.3.D_7.5 vs. 2.3.D_30	-19587	-52823 to 13649	No	ns	0.2720
2.3.D_15 vs. 2.3.D_30	-5368	-38605 to 27868	No	ns	0.9406

[a] Partial (non-sequential) Sum of Squares [b] Degrees of Freedom [c] Mean Squares [d] F ratios based on Degrees of Freedom. n = numerator, d = denominator [e] Difference from the control mean. Negative values indicate enhanced growth and positive values indicate reduced growth. [f] Confidence interval (CI) of difference from control [g] Not significant (ns) > 0.05, * $P \leq 0.05$, ** $P \leq 0.01$, *** $P \leq 0.001$, **** $P \leq 0.0001$.

Table S23. Results of two-way ANOVA and Tukey's test comparing LA4 control AFG_2 with each VOC combination treatment.

3.D ANOVA	SS (Type III)[a]	DF[b]	MS[c]	F (DFn, DFd)[d]	*P* value
Replicate	437657	2	218829	F (2, 6) = 1.590	0.2791
Treatment	2753268	3	917756	F (3, 6) = 6.670	0.0244
**3.D Tukey's**	**Mean Diff.[e]**	**95.00% CI of diff.[f]**	**Below threshold?**	**Summary[g]**	**Adjusted *P* Value**
Control vs. 3.D_5	1141	92.23 to 2189	Yes	*	0.0354
Control vs. 3.D_10	1111	62.90 to 2160	Yes	*	0.0394
Control vs. 3.D_20	1061	12.23 to 2109	Yes	*	0.0477
3.D_5 vs. 3.D_10	-29.33	-1078 to 1019	No	ns	0.9996
3.D_5 vs. 3.D_20	-80.00	-1128 to 968.4	No	ns	0.9929
3.D_10 vs. 3.D_20	-50.67	-1099 to 997.8	No	ns	0.9981
**2.D ANOVA**	**SS (Type III)[a]**	**DF[b]**	**MS[c]**	**F (DFn, DFd)[d]**	***P* value**
Replicate	30513	2	15257	F (2, 6) = 0.09246	0.9130
Treatment	2838338	3	946113	F (3, 6) = 5.734	0.0339
**2.D Tukey's**	**Mean Diff.[e]**	**95.00% CI of diff.[f]**	**Below threshold?**	**Summary[g]**	**Adjusted *P* Value**
Control vs. 2.D_5	1227	79.20 to 2375	Yes	*	0.0381
Control vs. 2.D_10	1039	-109.1 to 2187	No	ns	0.0735
Control vs. 2.D_20	1066	-82.14 to 2214	No	ns	0.0667
2.D_5 vs. 2.D_10	-188.3	-1336 to 959.8	No	ns	0.9381
2.D_5 vs. 2.D_20	-161.3	-1309 to 986.8	No	ns	0.9593
2.D_10 vs. 2.D_20	27.00	-1121 to 1175	No	ns	0.9998
**2.3 ANOVA**	**SS (Type III)[a]**	**DF[b]**	**MS[c]**	**F (DFn, DFd)[d]**	***P* value**
Replicate	49651	2	24826	F (2, 6) = 0.8313	0.4801
Treatment	4362754	3	1454251	F (3, 6) = 48.70	0.0001
**2.3 Tukey's**	**Mean Diff.[e]**	**95.00% CI of diff.[f]**	**Below threshold?**	**Summary[g]**	**Adjusted *P* Value**
Control vs. 2.3_5	1506	1018 to 1995	Yes	***	0.0002
Control vs. 2.3_10	1238	749.6 to 1726	Yes	***	0.0005
Control vs. 2.3_20	1381	892.6 to 1869	Yes	***	0.0003
2.3_5 vs. 2.3_10	-268.3	-756.8 to 220.1	No	ns	0.3190
2.3_5 vs. 2.3_20	-125.3	-613.8 to 363.1	No	ns	0.8114
2.3_10 vs. 2.3_20	143.0	-345.4 to 631.4	No	ns	0.7484
**2.3.D ANOVA**	**SS (Type III)[a]**	**DF[b]**	**MS[c]**	**F (DFn, DFd)[d]**	***P* value**
Replicate	127373	2	63687	F (2, 6) = 0.4603	0.6517
Treatment	2394818	3	798273	F (3, 6) = 5.770	0.0335
**2.3.D Tukey's**	**Mean Diff.[e]**	**95.00% CI of diff.[f]**	**Below threshold?**	**Summary[g]**	**Adjusted *P* Value**
Control vs. 2.3.D_7.5	1133	81.67 to 2184	Yes	*	0.0368
Control vs. 2.3.D_15	1040	-11.67 to 2091	No	ns	0.0523
Control vs. 2.3.D_30	822.0	-229.3 to 1873	No	ns	0.1224
2.3.D_7.5 vs. 2.3.D_15	-93.33	-1145 to 958.0	No	ns	0.9889
2.3.D_7.5 vs. 2.3.D_30	-311.0	-1362 to 740.3	No	ns	0.7429
2.3.D_15 vs. 2.3.D_30	-217.7	-1269 to 833.7	No	ns	0.8871

[a] Partial (non-sequential) Sum of Squares [b] Degrees of Freedom [c] Mean Squares. [d] F ratios based on Degrees of Freedom. n = numerator, d = denominator [e] Difference from the control mean. Negative values indicate enhanced growth and positive values indicate reduced growth. [f] Confidence interval (CI) of difference from control [g] Not significant (ns) > 0.05, * $P \leq 0.05$, ** $P \leq 0.01$, *** $P \leq 0.001$, **** $P \leq 0.0001$

Table S24. Results of two-way ANOVA and Tukey's test comparing LA4 control CPA with each VOC combination treatment.

3.D ANOVA	SS (Type III)[a]	DF[b]	MS[c]	F (DFn, DFd)[d]	*P* value
Replicate	118847	2	59424	F (2, 6) = 1.246	0.3527
Treatment	42508245	3	14169415	F (3, 6) = 297.1	< 0.0001
**3.D Tukey's**	**Mean Diff.[e]**	**95.00% CI of diff.[f]**	**Below threshold?**	**Summary[g]**	**Adjusted *P* Value**
Control vs. 3.D_5	4293	3676 to 4911	Yes	****	< 0.0001
Control vs. 3.D_10	4312	3695 to 4929	Yes	****	< 0.0001
Control vs. 3.D_20	4429	3812 to 5047	Yes	****	< 0.0001
3.D_5 vs. 3.D_10	18.67	-598.6 to 635.9	No	ns	0.9995
3.D_5 vs. 3.D_20	136.0	-481.2 to 753.2	No	ns	0.8683
3.D_10 vs. 3.D_20	117.3	-499.9 to 734.6	No	ns	0.9090
**2.D ANOVA**	**SS (Type III)[a]**	**DF[b]**	**MS[c]**	**F (DFn, DFd)[d]**	***P* value**
Replicate	25554	2	12777	F (2, 6) = 0.1133	0.8948
Treatment	41505294	3	13835098	F (3, 6) = 122.6	< 0.0001
**2.D Tukey's**	**Mean Diff.[e]**	**95.00% CI of diff.[f]**	**Below threshold?**	**Summary[g]**	**Adjusted *P* Value**
Control vs. 2.D_5	4363	3414 to 5312	Yes	****	<0.0001
Control vs. 2.D_10	4223	3273 to 5172	Yes	****	<0.0001
Control vs. 2.D_20	4295	3345 to 5244	Yes	****	<0.0001
2.D_5 vs. 2.D_10	-140.3	-1090 to 809.0	No	ns	0.9533
2.D_5 vs. 2.D_20	-68.33	-1018 to 881.0	No	ns	0.9940
2.D_10 vs. 2.D_20	72.00	-877.4 to 1021	No	ns	0.9930
**2.3 ANOVA**	**SS (Type III)[a]**	**DF[b]**	**MS[c]**	**F (DFn, DFd)[d]**	***P* value**
Replicate	73200	2	36600	F (2, 6) = 0.8704	0.4657
Treatment	53043981	3	17681327	F (3, 6) = 420.5	< 0.0001
**2.3 Tukey's**	**Mean Diff.[e]**	**95.00% CI of diff.[f]**	**Below threshold?**	**Summary[g]**	**Adjusted *P* Value**
Control vs. 2.3_5	4862	4282 to 5442	Yes	****	<0.0001
Control vs. 2.3_10	4819	4239 to 5398	Yes	****	<0.0001
Control vs. 2.3_20	4885	4305 to 5464	Yes	****	<0.0001
2.3_5 vs. 2.3_10	-43.33	-622.9 to 536.3	No	ns	0.9933
2.3_5 vs. 2.3_20	22.67	-556.9 to 602.3	No	ns	0.9990
2.3_10 vs. 2.3_20	66.00	-513.6 to 645.6	No	ns	0.9774
**2.3.D ANOVA**	**SS (Type III)[a]**	**DF[b]**	**MS[c]**	**F (DFn, DFd)[d]**	***P* value**
Replicate	2068945	2	1034472	F (2, 6) = 1.673	0.2646
Treatment	27905277	3	9301759	F (3, 6) = 15.04	0.0034
**2.3.D Tukey's**	**Mean Diff.[e]**	**95.00% CI of diff.[f]**	**Below threshold?**	**Summary[g]**	**Adjusted *P* Value**
Control vs. 2.3.D_7.5	3677	1455 to 5900	Yes	**	0.0050
Control vs. 2.3.D_15	480.3	-1742 to 2703	No	ns	0.8744
Control vs. 2.3.D_30	2708	485.2 to 4930	Yes	*	0.0216
2.3.D_7.5 vs. 2.3.D_15	-3197	-5419 to -974.6	Yes	**	0.0099
2.3.D_7.5 vs. 2.3.D_30	-969.7	-3192 to 1253	No	ns	0.4868
2.3.D_15 vs. 2.3.D_30	2227	4.885 to 4450	Yes	*	0.0496

[a] Partial (non-sequential) Sum of Squares [b] Degrees of Freedom [c] Mean Squares [d] F ratios based on Degrees of Freedom. n = numerator, d = denominator [e] Difference from the control mean. Negative values indicate enhanced growth and positive values indicate reduced growth. [f] Confidence interval (CI) of difference from control [g] Not significant (ns) > 0.05, * $P \leq 0.05$, ** $P \leq 0.01$, *** $P \leq 0.001$, **** $P \leq 0.0001$.

Table S25. Results of two-way ANOVA and Tukey's test comparing LA4 control IDTs with each VOC combination treatment.

3.D ANOVA	SS (Type III)[a]	DF[b]	MS[c]	F (DFn, DFd)[d]	*P* value
Replicate	513183	2	256592	F (2, 6) = 1.060	0.4034
Treatment	1290940	3	430313	F (3, 6) = 1.778	0.2511
**3.D Tukey's**	**Mean Diff.[e]**	**95.00% CI of diff.[f]**	**Below threshold?**	**Summary[g]**	**Adjusted *P* Value**
Control vs. 3.D_5	-770.7	-2161 to 619.8	No	ns	0.3129
Control vs. 3.D_10	-66.00	-1456 to 1324	No	ns	0.9982
Control vs. 3.D_20	16.33	-1374 to 1407	No	ns	> 0.9999
3.D_5 vs. 3.D_10	704.7	-685.8 to 2095	No	ns	0.3763
3.D_5 vs. 3.D_20	787.0	-603.4 to 2177	No	ns	0.2987
3.D_10 vs. 3.D_20	82.33	-1308 to 1473	No	ns	0.9966
**2.D ANOVA**	**SS (Type III)[a]**	**DF[b]**	**MS[c]**	**F (DFn, DFd)[d]**	***P* value**
Replicate	1541607	2	770804	F (2, 6) = 1.413	0.3142
Treatment	2800040	3	933347	F (3, 6) = 1.711	0.2634
**2.D Tukey's**	**Mean Diff.[e]**	**95.00% CI of diff.[f]**	**Below threshold?**	**Summary[g]**	**Adjusted *P* Value**
Control vs. 2.D_5	-668.3	-2756 to 1419	No	ns	0.6982
Control vs. 2.D_10	-1143	-3231 to 944.3	No	ns	0.3212
Control vs. 2.D_20	0.000	-2088 to 2088	No	ns	> 0.9999
2.D_5 vs. 2.D_10	-475.0	-2563 to 1613	No	ns	0.8577
2.D_5 vs. 2.D_20	668.3	-1419 to 2756	No	ns	0.6982
2.D_10 vs. 2.D_20	1143	-944.3 to 3231	No	ns	0.3212
**2.3 ANOVA**	**SS (Type III)[a]**	**DF[b]**	**MS[c]**	**F (DFn, DFd)[d]**	***P* value**
Replicate	685565	2	342783	F (2, 6) = 6.303	0.0335
Treatment	222551	3	74184	F (3, 6) = 1.364	0.3404
**2.3 Tukey's**	**Mean Diff.[e]**	**95.00% CI of diff.[f]**	**Below threshold?**	**Summary[g]**	**Adjusted *P* Value**
Control vs. 2.3_5	103.0	-556.2 to 762.2	No	ns	0.9457
Control vs. 2.3_10	-205.3	-864.5 to 453.8	No	ns	0.7142
Control vs. 2.3_20	-216.3	-875.5 to 442.8	No	ns	0.6832
2.3_5 vs. 2.3_10	-308.3	-967.5 to 350.8	No	ns	0.4352
2.3_5 vs. 2.3_20	-319.3	-978.5 to 339.8	No	ns	0.4093
2.3_10 vs. 2.3_20	-11.00	-670.2 to 648.2	No	ns	> 0.9999
**2.3.D ANOVA**	**SS (Type III)[a]**	**DF[b]**	**MS[c]**	**F (DFn, DFd)[d]**	***P* value**
Replicate	357708	2	178854	F (2, 6) = 3.954	0.0803
Treatment	305847	3	101949	F (3, 6) = 2.254	0.1825
**2.3.D Tukey's**	**Mean Diff.[e]**	**95.00% CI of diff.[f]**	**Below threshold?**	**Summary[g]**	**Adjusted *P* Value**
Control vs. 2.3.D_7.5	153.0	-448.1 to 754.1	No	ns	0.8149
Control vs. 2.3.D_15	352.3	-248.8 to 953.5	No	ns	0.2756
Control vs. 2.3.D_30	398.0	-203.1 to 999.1	No	ns	0.2019
2.3.D_7.5 vs. 2.3.D_15	199.3	-401.8 to 800.5	No	ns	0.6768
2.3.D_7.5 vs. 2.3.D_30	245.0	-356.1 to 846.1	No	ns	0.5368
2.3.D_15 vs. 2.3.D_30	45.67	-555.5 to 646.8	No	ns	0.9930

[a] Partial (non-sequential) Sum of Squares [b] Degrees of Freedom [c] Mean Squares [d] F ratios based on Degrees of Freedom. n = numerator, d = denominator [e] Difference from the control mean. Negative values indicate enhanced growth and positive values indicate reduced growth. [f] Confidence interval (CI) of difference from control [g] Not significant (ns) > 0.05, * $P \leq 0.05$, ** $P \leq 0.01$, *** $P \leq 0.001$, **** $P \leq 0.0001$.
